# A real-world pharmacovigilance study of mepolizumab in the FDA adverse event reporting system (FAERS) database

**DOI:** 10.3389/fphar.2023.1320458

**Published:** 2023-12-21

**Authors:** Fan Zou, Chengyu Zhu, Siyu Lou, Zhiwei Cui, Dan Wang, Yingyong Ou, Li Wang, Junyou Chen, Yuanbo Lan

**Affiliations:** ^1^ Department of Respiratory and Critical Care Medicine, Affiliated Hospital of Zunyi Medical University, Zunyi, China; ^2^ Department of Obstetrics and Gynecology, The First Affiliated Hospital of Xi’an Jiaotong University, Xi’an, China; ^3^ Department of Respiratory and Critical Care Medicine, The Second Affiliated Hospital of Xi’an Jiaotong University, Xi’an, China

**Keywords:** mepolizumab, adverse drug event, FAERS, real-world study, asthma

## Abstract

Mepolizumab is primarily used in the treatment of asthma, eosinophilic granulomatosis with polyangiitis, eosinophilia syndrome, and chronic rhinitis with nasal polyps. The information about its adverse drug reactions is mainly derived from clinical trials, and there is a shortage of real-world studies with extensive sample sizes. In this study, the U.S. FDA’s Adverse Event Reporting System (FAERS) database was analyzed to evaluate the side effects of mepolizumab. A total of 18,040 reports of mepolizumab-associated adverse events were identified from the FDA Adverse Event Reporting System database. Multiple disproportionality analysis algorithms were used to determine the significance of these AEs. The study identified 198 instances of mepolizumab-induced AEs, including some important AEs not mentioned in the product labeling. The time to onset of adverse reactions was also analyzed, with a median time of 109 days. Most AEs occurred within the first month of mepolizumab use, but some may still occur after 1 year of treatment. Gender-specific analysis showed different high-risk AEs for females (digestive and neurological side effects) and males (serious adverse effects leading to hospitalization and death). The findings mentioned provide valuable insights on optimizing the use of mepolizumab, enhancing its effectiveness, and minimizing potential side effects. This information will greatly contribute to the practical implementation of the drug in clinical settings.

## 1 Introduction

Asthma, a long-term inflammatory condition of the respiratory passages, is characterized by indications like coughing, wheezing, difficulty breathing, and tightness in the chest (Hammad and Lambrecht, 2021). It has a global impact, affecting approximately 300 million individuals of diverse ages and ethnic backgrounds, and tragically causing around 250,000 deaths annually. When individuals with asthma continue to experience uncontrolled symptoms despite receiving appropriate treatment, they are now recognized as having severe asthma, which imposes a significant financial burden on healthcare providers. As per the guidelines established by the European Respiratory Society (ERS) and the American Thoracic Society, severe asthma is characterized as asthma that necessitates the use of high-dose corticosteroid medication, along with another controller, to attain control, or asthma that persists uncontrolled despite this treatment (Chung et al., 2014). Approximately 5%–10% of asthma patients are believed to suffer from severe asthma, which places a significant burden on healthcare resources (Schoettler and Mary, 2020).

Eosinophilic inflammation in the airways is closely linked to the severity of asthma, with tissue and blood eosinophil counts directly influencing the frequency of asthma attacks and the risk of irreversible airway obstruction (Khalfaoui et al., 2022). The development, maturation, and survival of eosinophils in tissues are closely linked to disease severity and airway eosinophilia, with Interleukin-5 (IL-5) playing a vital role (Hassani and Koenderman, 2018). To target IL-5, a significant driver of eosinophilic inflammation, mepolizumab, a humanized monoclonal anti-IL-5 antibody, has been developed. The FDA has approved this medication as an additional maintenance treatment for severe asthma in patients aged 12 years and older, effectively decreasing blood eosinophil counts (approved by the FDA in November 2015) (Castillo et al., 2017). Mepolizumab has been approved in different parts of the world for treating eosinophilic granulomatosis with polyangiitis, hypereosinophilic syndrome, and chronic rhinosinusitis with nasal polyps (Pavord et al., 2022). Numerous randomized controlled experiments have shown that mepolizumab is a viable and easily tolerated choice for treatment. Studies have demonstrated that it decreases the occurrence of asthma flare-ups in individuals suffering from severe eosinophilic asthma, resulting in better management of symptoms and improved overall quality of life (Pavord et al., 2012; Ortega et al., 2014). Furthermore, mepolizumab has demonstrated the ability to decrease the size of polyps and relieve nasal blockage in individuals with chronic rhinosinusitis accompanied by nasal polyps, irrespective of the presence of asthma or Aspirin-exacerbated respiratory disease (Roufosse et al., 2020).

Despite the extensive use of mepolizumab in clinical settings, there has been a gradual increase in reports of related adverse events (AEs) (Corren, 2019; Aldajani et al., 2022). Injection site reactions, diarrhea, pruritus, headache, gastrointestinal disorders, musculoskeletal disorders, nasopharyngitis, sinusitis, bronchitis, and upper respiratory tract infections were frequently reported as treatment-emergent adverse events in phase II and phase III clinical trials of mepolizumab. Several severe adverse events were documented, such as deterioration of symptoms related to hypereosinophilic syndrome, infection caused by M.abscessus, eosinophilic gastroenteritis, and peripheral T-cell lymphoma. This information was reported by F. Roufosse et al. in a phase III, randomized, placebo-controlled trial assessing the efficacy and safety of mepolizumab in hypereosinophilic syndrome (Gleich et al., 2021). Nevertheless, the effectiveness and safety information for mepolizumab over an extended period has primarily been documented through case reports, clinical trials, and meta-analyses (Henriksen et al., 2018; Domingo Ribas et al., 2021). The research has concentrated on particular systems or included relatively limited sample sizes and specific criteria for selection. As a result, comprehensive safety data from large samples and real-world cohorts are currently lacking. To assess the safety of mepolizumab in real-world scenarios, this pharmacovigilance analysis was performed due to the extensive clinical utilization and the necessity for adverse event evaluations.

The FAERS database, which is open to the public, is a spontaneous reporting system (SRS) that contains a wide range of case reports documenting adverse drug events. These reports are submitted by healthcare professionals, pharmacists, manufacturers, and other individuals(Yu et al., 2021). FAERS, being the biggest worldwide pharmacovigilance repository, functions as a valuable resource for detecting adverse events linked to drug usage(Fusaroli et al., 2022). The aim of this research was to assess the AEs of mepolizumab by analyzing post-marketing data from FAERS. Our main objective in these findings is to offer valuable perspectives for clinical surveillance and the detection of possible hazards linked to mepolizumab.

## 2 Materials and methods

### 2.1 Data source

FAERS, also known as the FDA Adverse Event Reporting System, is a comprehensive database where adverse event reports, prescription errors, and complaints regarding product quality that have led to AEs are stored. More information about FAERS can be found at https://www.fda.gov/drugs/questions-and-answers-fdas-adverse-event-reporting-system-faers. The database aids in the FDA’s monitoring of the safety of pharmaceutical and therapeutic biologic products after they have been approved for marketing. The FAERS database consists of seven datasets that cover different types of data, including patient demographic and administrative information (DEMO), drug information (DRUG), adverse event coding (REAC), patient outcomes (OUTC), report sources (RPSR), therapy start and end dates for reported drugs (THER), and indications for drug administration (INDI).

**FIGURE 1:**
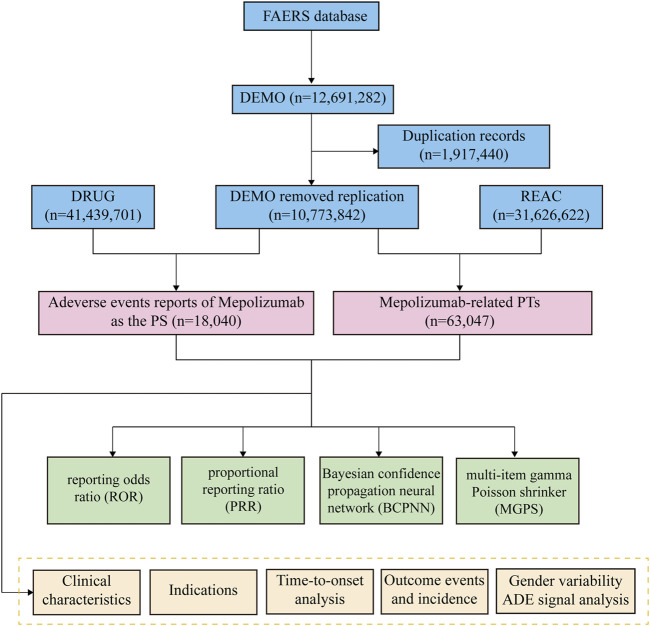
The process of selecting mepolizumab-associated AEs from FAERS database.

The research included the examination of AEs information associated with mepolizumab, which was acquired from the FAERS database. Data extraction was performed from the fourth quarter of 2015 (2015 Q4) through the first quarter of 2023 (2023 Q1). The Statistical Analysis System (SAS) 9.4 was utilized for data gathering and preprocessing. Initially, the FAERS database yielded a grand total of 12,691,282 reports. Nevertheless, because of the regular updates of the database, it became imperative to reanalyze the data to remove any redundant instances of previous public reports. Before conducting statistical analysis, a deduplication procedure was carried out in accordance with the guidelines provided by the FDA. To accomplish this, the most recent FDA_DT was chosen when the CASEID values were identical, and the PRIMARYID with a higher value was selected when both CASEID and FDA_DT were a match (Shu et al., 2022a; Shu et al., 2022b). After going through this deduplication procedure, incomplete, incorrect, and duplicate reports were excluded and the total count of reports decreased to 10,773,842. Both the trademarks and generic names were utilized to identify records associated with etoposide. The search involved the use of ‘Mepolizumab’ and ‘NUCALA’ in this particular study. The drugs reported in FAERS were categorized into four modalities: PS (primary suspect), SS (second suspect), C (concomitant), and I (interacting). To enhance the precision of the analyses and eliminate the influence of concurrent medications, the AEs role code was preserved exclusively for instances where the PS drug was identified as ‘mepolizumab’ (Zhang et al., 2023). The highest level of terminology used for coding all AEs in the report is the System Organ Class (SOC) based on the Medical Dictionary for Regulatory Activities (MedDRA, version 26.0). We screened a grand total of 63,047 terms related to mepolizumab, which were categorized as preferred terms (PTs). During the period of this research, we identified totally 18,040 AEs reports of etoposide as the PS drug. To reduce confounding, in the disproportionality analysis at PT level, we removed PTs associated with the mepolizumab indication (Tang et al., 2022). Figure 1 displays the flow chart of the investigation.

### 2.2 Statistical analysis

Disproportionate analysis is a tool for hypothesizing possible causal relationships between drugs and adverse reactions, with subsequent clinical assessment of underlying case reports (Caster et al., 2020). It is based on a comparison of the observed and expected number of reports for any given combination of drug and adverse event and is often recommended for vigilance analyses of adverse drug reactions in large spontaneous reporting databases (Montastruc et al., 2011). Reporting odds ratio (ROR), proportional reporting ratio (PRR), Bayesian confidence propagation neural network (BCPNN) and Multi-item gamma Poisson shrinker (MGPS) are common algorithms for disproportionality analysis and are currently widely used by the Healthcare Products Regulatory Agency (MHRA), the Netherlands Pharmacovigilance Centre, the World Health Organization (WHO) and the FDA (Sakaeda et al., 2013). The ROR and PRR algorithms are frequentist (non-Bayesian) algorithms, and the advantage of ROR is that it corrects for bias due to the low number of reports of certain events compared to PRR (Rothman et al., 2004). The advantage of PRR over ROR is that it is less affected by omission of adverse events (Evans et al., 2001). In conclusion, the non-Bayesian method (frequency method) is simple to calculate and has high sensitivity, but when the number of adverse events is small, the likelihood of false positives is high (Wu et al., 2023). BCPNN and MGPS algorithms are Bayesian algorithms. BCPNN is excellent in integrating data from multiple sources and cross validation, MGPS has the advantage that it is able to detect signals from rare events (Bate et al., 1998; Kubota et al., 2004). The Bayesian approach is stable. It accounts for the uncertainty in the disproportionate rate when the reports are small, reduces the likelihood of false positives, and is used for pattern recognition in higher dimensions, but it is computationally complex and has a relatively lagged signal detection time (Tang et al., 2022). Therefore, this study adopts the joint use of multiple algorithms, makes reasonable use of the advantages of different algorithms, expands the detection range, and verifies the results from multiple perspectives in order to detect more comprehensive and reliable safety signals (Sakaeda et al., 2013; Noguchi et al., 2018; Zhou et al., 2023). PTs with reported counts ≥3 were selected for the initial screening in this study (Jiang et al., 2023). The signal detection thresholds for each algorithm are set according to authoritative methods (Bate et al., 1998; Evans et al., 2001; Szarfman et al., 2002; van Puijenbroek et al., 2002), and the specific formulas and thresholds are detailed in Table 1.

**TABLE 1:** The specific formulas for the four algorithms are as follows. Notes: Equation: a, number of reports containing both the target drug and the target adverse drug reaction; b, number of reports containing other adverse drug reactions of the target drug; c, number of reports containing the target adverse drug reaction of other drugs; d, number of reports containing other drugs and other adverse drug reactions. The MGPS employs an empirical Bayesian approach, whereby a prior distribution is obtained by maximum likelihood estimates, and the prior and likelihood are subsequently combined to obtain a posterior distribution. The fifth percentile of the posterior distribution is denoted by “EBGM05” and is interpreted as the one-sided 95% confidence lower bound for the EBGM. Abbreviations: 95% CI, 95% confidence interval; N, the number of reports; χ2, chi-squared; IC, information component; IC025, the lower limit of the 95% CI of the IC; E (IC), the IC expectations; V (IC), the variance of IC; EBGM, empirical Bayesian geometric mean; EBGM05, empirical Bayesian geometric mean lower 95% CI for the posterior distribution.

Algorithms	Equation	Criteria
ROR	ROR = ad/bc	Lower limit of 95% CI > 1, N ≥ 3
	95%CI = e^ln(ROR)±1.96(1/a+1/b+1/c+1/d)^0.5^	
PRR	PRR = [a(c+d)]/[c(a+b)]	PRR ≥ 2, χ^2^ ≥ 4, N ≥ 3
	χ^2^ = [(ad-bc)^2](a+b+c+d)/[(a+b)(c+d)(a+c)(b+d)]	
BCPNN	IC = log_2_a(a+b+c+d)/[(a+c)(a+b)]	IC025 > 0
	95%CI = E(IC) ± 2[V(IC)]^0.5	
MGPS	EBGM = a(a+b+c+d)/[(a+c)(a+b)]	EBGM05 > 2
	95%CI = e^ln(EBGM)±1.96(1/a+1/b+1/c+1/d)^0.5^	

Additionally, the time to onset (TTO) of mepolizumab-induced AEs was defined as the interval between EVENT_DT (date of onset of AEs, in the DEMO file) and START_DT (date of initiation of mepolizumab, in the THER file). Deleted data include inaccurate or missing date inputs and EVENT_DT being earlier than START_DT.

Microsoft EXCEL 2019, SAS 9.4 (2013; SAS Institute Inc., Cary, North Carolina, United States), R software (version 4.2.1) are primarily employed for data processing and analysis. We used the “ggplot2” package in the R software for data visualization.

## 3 Results

### 3.1 Descriptive analysis

**FIGURE 2:**
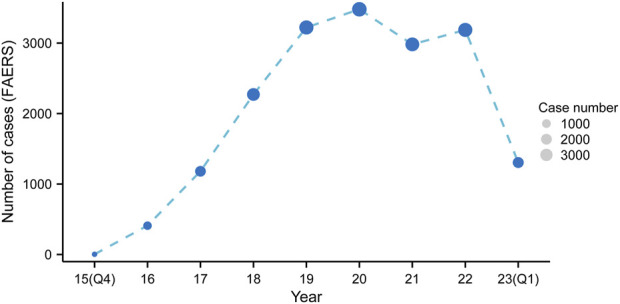
The annual distribution of mepolizumab-related AEs reports from 2015 to 2023.

Upon eliminating duplicates, a grand total of 18,040 adverse event reports were discovered, wherein mepolizumab was classified as the primary suspect drug. These reports corresponded to a collection of 63,047 mepolizumab-related preferred terms (PTs) (Figure 1). From 2015 to 2022, there was a steady rise in the reporting of AEs associated with mepolizumab, with the latest available information being the data for the first quarter of 2023 (Figure 2).

The AEs reported for mepolizumab are presented in Table 2, showcasing their characteristics. The largest proportion of reports (1.51%) originated from the elderly population (aged >64 years), while females (55.76%) accounted for a higher proportion compared to males (26.21%). The majority of reported weights were around 80 kg (3.94%). The majority of reports (73.69%) were provided by consumers, with health professionals accounting for around a quarter of the submissions (25.14%). In terms of geography, America had the largest percentage of reports (53.92%), with Canada (27.31%), Japan (2.87%), the United Kingdom (2.68%), and Australia (2.54%) following closely behind. Among the reported outcomes, serious outcomes (56.38%) were the most frequently documented, followed by hospitalization (34.18%) and death (7.55%). In 25.66% of cases, the utilization of Mepolizumab for unspecified purposes was documented, with asthma (64.72%) being the most frequently reported indication.

**TABLE 2:** Clinical characteristics of reports with mepolizumab from the FAERS database.

Characteristics	Case number, n	Case Proportion, %
**Number of events**	18,040	
**Age**		
<18	6	0.03
18–64	187	1.04
>64	273	1.51
Unknown	17,574	97.42
**Gender**		
Female	10,060	55.76
Male	4,728	26.21
Unknown	3,252	18.03
**Weight**		
<80	710	3.94
80–100	363	2.01
>100	220	1.22
Unknown	16,747	92.83
**Reported Person**		
Health professional	4,536	25.14
Consumer	13,295	73.69
Unknown	209	11.57
**Reported Countries (top five)**		
America	9,728	53.92
Canada	4,927	27.31
Japan	517	2.87
United Kiongdom	483	2.68
Australia	458	2.54
**Serious Outcomes**	n = 14,110	
Death (DE)	1,066	7.55
Life-threatening (LF)	152	1.08
Hospitalization (HO)	4,823	34.18
Disability (DS)	106	0.75
Other serious outcomes	7,955	56.38
Unknown	8	0.06
**Indications (top five)**		
Asthma	11,676	64.72
Product used for unknown indication	4,665	25.86
Eosinophilic granulomatosis with polyangiitis	542	3.00
Hypereosinophilic syndrome	121	0.67
Nasal polyps	89	0.49

### 3.2 Signal of system organ class

Table 3 presents the signal intensities of mepolizumab-associated AEs categorized by SOCs. A total of 27 organ systems were impacted by adverse events associated with mepolizumab, as indicated by our statistical analysis. Among these, several significant SOCs were identified based on meeting the criteria of at least one of the four indices used for analysis. The significant SOCs included respiratory, thoracic, and mediastinal disorders (case = 12,574, ROR 5.20[95%CI 5.10–5.30]); general disorders and administration site conditions (case = 11,309, ROR 1.01[95%CI 0.99–1.03]); injury, poisoning, and procedural complications (case = 8,185, ROR 1.16[95%CI 1.13–1.19]); infections and infestations (case = 6,366, ROR 1.97[95%CI 1.92–2.02]); surgical and medical procedures (case = 1726, ROR 2.06[95%CI 1.96–2.16]); immune system disorders (case = 894, ROR 1.17[95%CI 1.10–1.25]); and social circumstances(case = 752, ROR 2.66[95%CI 2.47–2.86]). These findings highlight the specific organ systems where mepolizumab-induced AEs were most frequently reported and indicate areas that warrant further attention and investigation.

**TABLE 3:** Signal strength of reports of mepolizumab at the System Organ Class (SOC) level in FAERS database. Notes: Red are those that follow the algorithm.

System Organ Class (SOC)	Case Numbers	ROR (95% Two-Sided CI)	PRR	χ2	IC (IC025)	EBGM(EBGM05)
Respiratory, thoracic and mediastinal disorders	12,574	5.20(5.10–5.30)	4.36	33,876.31	2.12(0.45)	4.33(4.26)
General disorders and administration site conditions	11,309	1.01(0.99–1.03)	1.01	1.09	0.01(−1.65)	1.01(0.99)
Injury, poisoning and procedural complications	8,185	1.16(1.13–1.19)	1.14	153.85	0.19(−1.48)	1.14(1.12)
Infections and infestations	6,366	1.97(1.92–2.02)	1.87	2714.94	0.90(−0.77)	1.87(1.83)
Nervous system disorders	3,432	0.68(0.66–0.70)	0.7	489.38	−0.52(−2.19)	0.70(0.68)
Musculoskeletal and connective tissue disorders	3,034	0.92(0.88–0.95)	0.92	22.73	−0.12(−1.79)	0.92(0.89)
Investigations	2,525	0.67(0.64–0.70)	0.68	398.91	−0.55(−2.22)	0.68(0.66)
Skin and subcutaneous tissue disorders	2,482	0.67(0.65–0.70)	0.69	374.6	−0.54(−2.21)	0.69(0.66)
Gastrointestinal disorders	2369	0.43(0.41–0.45)	0.45	1715.95	−1.14(−2.81)	0.45(0.44)
Surgical and medical procedures	1726	2.06(1.96–2.16)	2.03	912.07	1.02(−0.65)	2.03(1.95)
Psychiatric disorders	1,360	0.39(0.37–0.41)	0.40	1264.2	−1.31(−2.97)	0.40(0.39)
Cardiac disorders	971	0.74(0.70–0.79)	0.75	86.09	−0.42(−2.09)	0.75(0.71)
Immune system disorders	894	1.17(1.10–1.25)	1.17	22.74	0.23(−1.44)	1.17(1.11)
Product issues	888	0.82(0.77–0.88)	0.83	32.99	−0.27(−1.94)	0.83(0.78)
Eye disorders	790	0.65(0.60–0.69)	0.65	151.4	−0.62(−2.29)	0.65(0.61)
Social circumstances	752	2.66(2.47–2.86)	2.64	765.33	1.40(−0.27)	2.63(2.48)
Vascular disorders	740	0.60(0.56–0.64)	0.6	196.54	−0.73(−2.39)	0.60(0.57)
Neoplasms benign, malignant and unspecified (incl cysts and polyps)	679	0.32(0.30–0.35)	0.33	958.4	−1.60(−3.27)	0.33(0.31)
Metabolism and nutrition disorders	536	0.41(0.38–0.45)	0.42	439.58	−1.25(−2.92)	0.42(0.39)
Renal and urinary disorders	419	0.32(0.29–0.36)	0.33	591.42	−1.61(−3.28)	0.33(0.30)
Blood and lymphatic system disorders	260	0.25(0.22–0.29)	0.26	567.39	−1.96(−3.62)	0.26(0.23)
Ear and labyrinth disorders	238	0.86(0.76–0.98)	0.87	4.99	−0.21(−1.87)	0.87(0.78)
Hepatobiliary disorders	153	0.30(0.26–0.35)	0.30	250.67	−1.73(−3.40)	0.30(0.26)
Reproductive system and breast disorders	143	0.29(0.25–0.34)	0.29	247.74	−1.78(−3.44)	0.29(0.25)
Endocrine disorders	119	0.73(0.61–0.88)	0.73	11.56	−0.45(−2.11)	0.73(0.63)
Congenital, familial and genetic disorders	58	0.34(0.26–0.44)	0.34	74.05	−1.55(−3.22)	0.34(0.28)
Pregnancy, puerperium and perinatal conditions	45	0.18(0.13–0.24)	0.18	168.85	−2.47(−4.14)	0.18(0.14)

### 3.3 Signal of preferred terms and subgroup analysis

All the four algorithms combined identified a total of 198 cases of AEs caused by mepolizumab, encompassing 20 System Organ Classes (SOCs) as shown in Supplementary Table S1. Table 4 presents a summary of reported PTs with a minimum of 20 occurrences. This table includes 63 PTs, corresponding to 11 SOCs. Importantly, our data mining revealed several significant AEs that were not explicitly mentioned in the mepolizumab product label. The unexpected AEs consist of PTs such as discharge of fluids, nonspecific response, recurrence of symptoms, discomfort in the chest, incomplete effectiveness of the therapeutic product, multiple allergies, infected sputum, COVID-19 infection, pneumonia, chronic inflammation of the sinuses, inflammation of the nasal passages, infection caused by *pseudomonas*, suspected COVID-19, exposure through contact with the skin, accidental exposure to the product, issue of missing product dose, inadequate dosage, reduced peak expiratory flow rate, abnormal count of eosinophils, increased level of immunoglobulin E in the blood, abnormal breathing sounds, abnormal oxygen saturation, reduced results of pulmonary function tests, abnormal complete blood count, increased respiratory rate, loss of sense of smell, sleep disorder due to a general medical condition, severe asthma attack, discolored sputum, congestion in the lungs, increased production of sputum, pain in the lungs, cough syndrome in the upper airways, sensation of choking, chronic obstructive pulmonary disease (COPD), congestion in the sinuses, disorder in the sinuses, abnormal lung sounds, loss of independence in daily activities, isolation of the patient, quarantine, sinus surgery, emergency medical care, hospitalization, and cataract surgery. Our analysis has identified additional AEs that emphasize and enhance the overall comprehension of mepolizumab’s safety profile.

**TABLE 4:** Signal strength of reports of mepolizumab at the PT level in the FAERS database. Notes: *, AEs that are not mentioned in the drug label. PT, Preferred Terms.

SOC Name	Preferred terms (PTs)	Case Numbers	ROR(95%Cl)	PRR	χ2	IC (IC025)	EBGM (EBGM05)
General disorders and administration site conditions	Secretion discharge*	117	8.43(7.02–10.12)	8.41	751.95	3.05(1.39)	8.29(7.12)
	Nonspecific reaction*	27	5.89(4.03–8.60)	5.88	108.21	2.54(0.88)	5.83(4.24)
	Symptom recurrence*	31	5.44(3.82–7.75)	5.44	111.08	2.43(0.76)	5.39(4.01)
	Ill-defined disorder	219	4.48(3.92–5.12)	4.47	584.14	2.15(0.48)	4.43(3.97)
	Chest discomfort*	411	4.23(3.84–4.66)	4.21	998.29	2.06(0.40)	4.18(3.85)
	Therapeutic product effect incomplete*	510	3.33(3.05–3.64)	3.31	820.21	1.72(0.06)	3.30(3.07)
	Polyp	23	3.24(2.15–4.89)	3.24	35.43	1.69(0.02)	3.23(2.29)
Immune system disorders	Multiple allergies*	46	5.60(4.19–7.49)	5.6	171.7	2.47(0.80)	5.54(4.35)
Infections and infestations	Sputum purulent*	29	40.12(27.48–58.58)	40.11	1023.8	5.22(3.55)	37.21(27.11)
	Coronavirus infection*	78	7.16(5.72–8.95)	7.15	406.92	2.82(1.15)	7.06(5.86)
	Respiratory tract infection	158	5.88(5.02–6.88)	5.87	630.69	2.54(0.87)	5.81(5.09)
	Pneumonia*	1,654	4.90(4.67–5.15)	4.8	4957.5	2.25(0.59)	4.76(4.57)
	Chronic sinusitis*	25	6.40(4.32–9.50)	6.4	112.52	2.66(1.00)	6.33(4.55)
	Herpes zoster	266	4.27(3.79–4.82)	4.26	658.64	2.08(0.42)	4.23(3.83)
	Lower respiratory tract infection	203	4.03(3.51–4.63)	4.02	457.34	2.00(0.33)	4.00(3.56)
	Rhinitis*	37	4.45(3.22–6.16)	4.45	98.16	2.14(0.48)	4.42(3.37)
	*Pseudomonas* infection*	33	4.23(3.00–5.96)	4.23	80.64	2.07(0.40)	4.20(3.15)
	Suspected COVID-19*	24	3.95(2.64–5.90)	3.95	52.43	1.97(0.31)	3.93(2.80)
	Viral upper respiratory tract infection	27	3.67(2.51–5.35)	3.67	51.95	1.87(0.20)	3.65(2.66)
Injury, poisoning and procedural complications	Exposure via skin contact*	600	151.10(137.88–165.58)	149.67	68,217.54	6.85(5.18)	115.45(106.94)
	Wrong technique in device usage process	560	10.41(9.57–11.32)	10.33	4625.01	3.34(1.68)	10.14(9.45)
	Accidental exposure to product*	578	5.73(5.27–6.22)	5.68	2209.48	2.49(0.83)	5.63(5.26)
	Product dose omission issue*	2170	3.67(3.52–3.84)	3.58	4050.28	1.83(0.17)	3.56(3.44)
	Product preparation issue	30	4.59(3.20–6.57)	4.59	83.41	2.19(0.52)	4.55(3.37)
	Underdose*	306	3.35(2.99–3.74)	3.33	497.49	1.73(0.06)	3.32(3.02)
Investigations	Peak expiratory flow rate decreased*	22	57.99(37.29–90.17)	57.97	1103.87	5.70(4.02)	52.06(35.98)
	Eosinophil count abnormal*	31	33.11(23.02–47.62)	33.09	904.96	4.96(3.29)	31.10(22.94)
	Coronavirus test positive*	51	29.57(22.30–39.23)	29.55	1328.47	4.81(3.14)	27.96(22.07)
	Eosinophil count decreased*	41	23.91(17.48–32.71)	23.9	858.47	4.51(2.85)	22.85(17.58)
	Blood immunoglobulin E increased*	36	13.26(9.52–18.46)	13.25	397.29	3.69(2.03)	12.94(9.81)
	Breath sounds abnormal*	47	9.50(7.12–12.68)	9.5	350.62	3.22(1.56)	9.34(7.33)
	Oxygen saturation abnormal*	24	7.49(5.00–11.21)	7.49	132.89	2.89(1.22)	7.39(5.27)
	Pulmonary function test decreased*	31	5.51(3.86–7.84)	5.5	113.01	2.45(0.78)	5.45(4.06)
	Full blood count abnormal*	173	4.62(3.98–5.37)	4.61	484.74	2.19(0.53)	4.58(4.04)
	Respiratory rate increased*	26	3.34(2.27–4.92)	3.34	42.37	1.73(0.07)	3.33(2.41)
Nervous system disorders Product issues	Anosmia*	35	3.38(2.42–4.71)	3.37	58.11	1.75(0.08)	3.36(2.54)
	Product complaint	646	23.12(21.36–25.03)	22.89	12,940.72	4.46(2.79)	21.94(20.53)
	Product availability issue	74	3.42(2.72–4.29)	3.41	125.38	1.76(0.10)	3.40(2.80)
Psychiatric disorders	Sleep disorder due to a general medical condition*	282	19.56(17.36–22.04)	19.48	4758.68	4.23(2.57)	18.78(17.00)
Respiratory, thoracic and mediastinal disorders	Asthmatic crisis*	474	114.39(103.51–126.43)	113.54	43,103.42	6.54(4.87)	92.74(85.29)
	Sputum discoloured*	203	18.03(15.67–20.74)	17.97	3141.96	4.12(2.45)	17.39(15.46)
	Pulmonary congestion*	97	8.51(6.97–10.41)	8.5	631.61	3.07(1.40)	8.38(7.08)
	Sputum increased*	21	8.87(5.76–13.65)	8.86	143.98	3.13(1.46)	8.73(6.08)
	Pulmonary pain*	30	7.90(5.51–11.34)	7.9	178.02	2.96(1.30)	7.79(5.76)
	Nasal congestion	248	4.17(3.68–4.73)	4.16	590.46	2.05(0.38)	4.13(3.72)
	Upper-airway cough syndrome*	43	4.63(3.43–6.25)	4.63	121.21	2.20(0.53)	4.60(3.57)
	Choking sensation*	24	4.63(3.10–6.92)	4.63	67.61	2.20(0.53)	4.59(3.28)
	Chronic obstructive pulmonary disease*	186	3.71(3.21–4.28)	3.7	364.1	1.88(0.21)	3.68(3.26)
	Sinus congestion*	49	3.91(2.95–5.18)	3.91	105.18	1.96(0.29)	3.88(3.07)
	Sinus disorder*	76	3.53(2.82–4.42)	3.53	136.7	1.81(0.15)	3.51(2.91)
	Rales*	23	3.98(2.64–5.99)	3.97	50.81	1.98(0.32)	3.95(2.80)
	Oropharyngeal discomfort	35	3.63(2.60–5.06)	3.63	66.1	1.85(0.18)	3.61(2.73)
	Dyspnoea	2,490	4.60(4.42–4.79)	4.46	6,680.8	2.15(0.48)	4.43(4.28)
	Rhinitis allergic	21	5.10(3.32–7.84)	5.1	68.55	2.34(0.67)	5.06(3.53)
	Bronchospasm	93	7.51(6.12–9.22)	7.5	516.58	2.89(1.22)	7.41(6.24)
Social circumstances	Social problem	116	33.06(27.39–39.89)	33	3376.76	4.96(3.29)	31.02(26.50)
	Loss of personal independence in daily activities*	472	6.06(5.53–6.64)	6.02	1954.58	2.58(0.91)	5.96(5.52)
Surgical and medical procedures	Patient isolation*	28	76.63(51.48–114.07)	76.6	1811.94	6.06(4.38)	66.57(47.72)
	Quarantine*	23	60.95(39.53–93.96)	60.92	1208.56	5.77(4.09)	54.42(37.88)
	Sinus operation*	45	19.22(14.27–25.89)	19.21	747.95	4.21(2.54)	18.53(14.45)
	Emergency care*	21	6.21(4.04–9.55)	6.21	90.6	2.62(0.95)	6.14(4.28)
	Hospitalisation*	614	3.53(3.26–3.82)	3.5	1093.89	1.80(0.14)	3.49(3.26)
	Cataract operation*	20	3.95(2.54–6.13)	3.95	43.63	1.97(0.30)	3.92(2.71)

We then conducted subgroup analyses, which can to some extent reduce the confounding of the results by demographic characteristics (de Vries et al., 2020). Among the two subgroups aged 18–64 and >64 years, the PT with the highest number of reported cases was product dose omission issue (subgroup ages <18 was exclude because of insufficient case reports). Additionally, when analyzing the top 15 reported AEs in each subgroup, we found that signals reported only among 18–64 subgroup included “condition aggravated”, “urticaria”, “chest pain”, “device use error”, and “sinusitis”. On the other hand, “malaise”, “cough”, “Inappropriate schedule of product administration”, “wheezing”, and “blood pressure increased” appeared to be more common in ages>64 subgroup (Supplementary Figure S1).

Similarly, subgroup analyses were performed for gender (Supplementary Figure S2), weight (Supplementary Figure S3), and reported person (Supplementary Figure S4) to analyze and compare similarities and differences in signals across subgroups. This information is essential for more refined clinical management, guiding clinical decision makers to adjust treatments based on the characteristics of specific subgroups.

### 3.4 Time to onset of mepolizumab-associated adverse events

**FIGURE 3:**
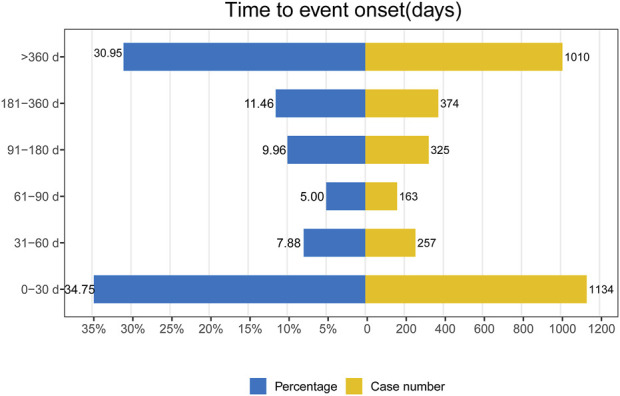
Time to onset of mepolizumab-related AEs.

The provided database furnished us with data concerning the initiation periods of adverse events associated with mepolizumab. Out of all the reported adverse events, a grand total of 3,263 included comprehensive and precise details regarding the time of occurrence. The AEs had a median onset time of 109 days, with an IQR of 7–469 days. In Figure 3, it can be seen that most AEs (1,134 or 34.75%) happened within the initial month of mepolizumab usage, as shown by the distribution of AE onset times. AEs were least likely to occur during the second to third month of treatment, with rates of 7.88% and 5% respectively, but significantly rose afterwards. Significantly, our data revealed that a considerable 30.95% of AEs remained possible following a year of mepolizumab treatment. These findings emphasize the importance of monitoring patients for potential AEs throughout the course of mepolizumab therapy, even beyond the initial months.

### 3.5 Signal of preferred terms gender difference risk

**FIGURE 4:**
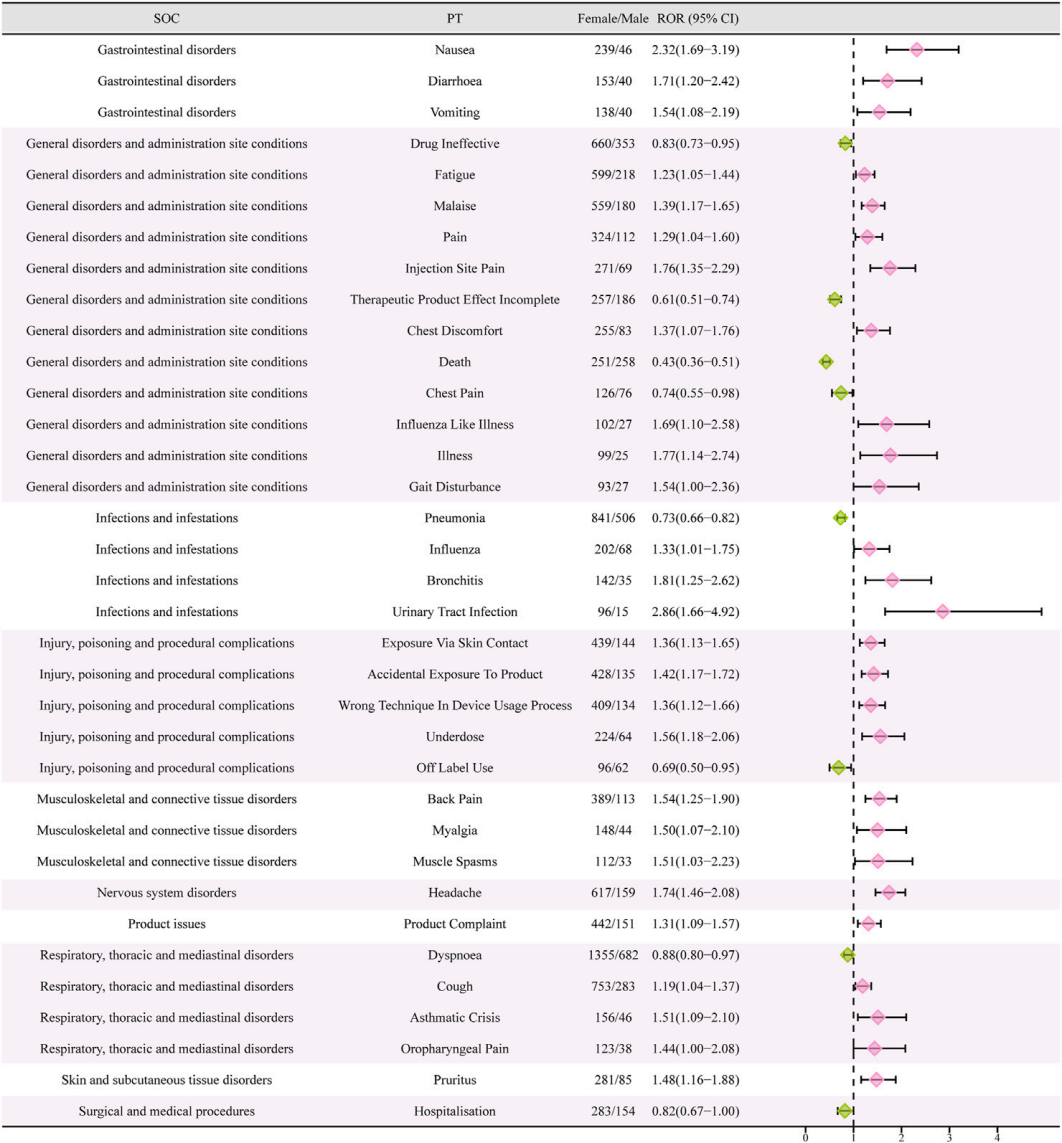
Reporting odds ratios (ROR) with 95% CI for all positive gender-related AEs. CI, confidence interval. The ROR here is not a strictly defined ROR in pharmacoepidemiological perspective.

Females who have symptoms like queasiness, diarrhea, throwing up, exhaustion, discomfort, infection site discomfort, chest uneasiness, flu-like sickness, walking difficulty, flu, bronchitis, urinary tract infection, exposure through skin contact, back discomfort, muscle pain, muscle cramp, head pain, cough, asthma attack, throat pain, itching, and more, were found to have high-risk signals during the signal detection analysis conducted at the PT level. In contrast, males had high-risk indicators that encompassed drug inefficacy, inadequate therapeutic outcomes, death, chest discomfort, lung infection, unapproved usage, difficulty breathing, and admission to the hospital (Figure 4).

**FIGURE 5:**
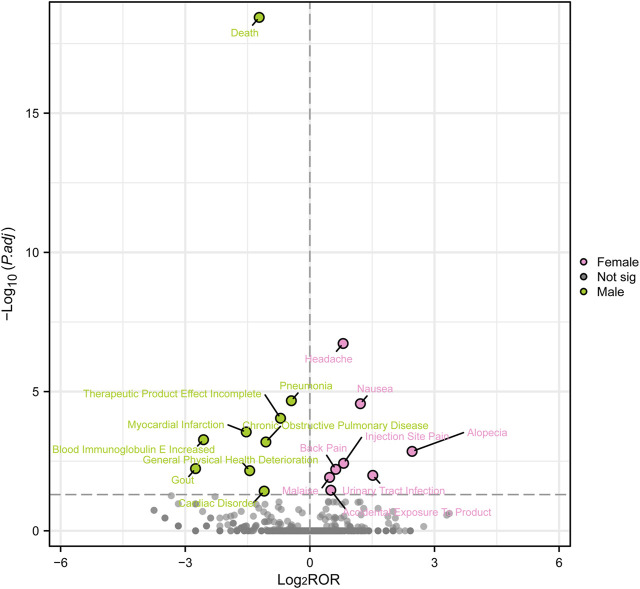
Volcanic map of gender difference risk signal for mepolizumab. ROR, reporting odds ratios; *P*.adj, the *p*-value is adjusted with false discovery rate (FDR) method.

In order to examine gender disparities in the findings of adverse event signal mining for mepolizumab, a visual representation known as a ‘volcano map’ was employed. The volcano map uses the -Log10*p*-value scale on the vertical axis and the Log2ROR value scale on the horizontal axis. Every point on the map indicates a pairing of the medication and negative reaction. Pink dots indicate potential adverse event signals in female patients, while green dots indicate potential adverse event signals in male patients. Furthermore, Figure 5 highlights significant adverse event signals that exhibit noteworthy Log2ROR and -Log10*p* values. The visual depictions offer valuable information on potential adverse event signals specific to gender related to mepolizumab, emphasizing the variations in reported AEs among males and females.

## 4 Discussion

Due to the scarcity of preclinical data, it is essential to gather pharmacovigilance data from post-marketing systems that report adverse events, which would greatly enhance drug specifications. Furthermore, it should be emphasized that information obtained from clinical trials may not precisely depict the actual circumstances in the real world, which encompasses a wide range of patients and comorbidities. The examination showed a consistent rise in the quantity of documented adverse events in recent times (Figure 2), possibly as a result of the increasing utilization of mepolizumab. The results highlight the significance of ongoing surveillance for adverse events. Based on our current understanding, this study on adverse events related to mepolizumab using the FAERS database is the most extensive pharmacovigilance investigation. It offers a comprehensive and methodical overview of worldwide reports regarding mepolizumab-associated adverse events in FAERS.

Based on the information from the baseline profile, it was observed that females (55.76%) experienced a higher occurrence of negative responses to mepolizumab in comparison to males (26.21%), which is consistent with asthma epidemiological research. Additionally, adverse reactions were less common in individuals below the age of 18 receiving mepolizumab. These observations are consistent with the primary target population of mepolizumab, which is additional treatment for patients with poorly controlled asthma. It is worth noting that patients with asthma before the age of 10 have a higher likelihood (up to 60%) of achieving asthma remission, whereas the remission rate in adults with asthma ranges from 5% to 15%(De Marco et al., 2002; Rönmark et al., 2007). Moreover, the higher prevalence of women among adults experiencing severe asthma could be attributed to the greater abundance of ILC2 in female individuals with asthma compared to their male counterparts(Cephus et al., 2017; Porsbjerg et al., 2023). Increased levels of type 2 innate lymphoid cells (ILC2 cells) may contribute to an intense allergic airway inflammation, resulting in insufficient management of asthma symptoms. Our reported findings indicate that mepolizumab is mainly linked to adverse events in female individuals, which is consistent with this observation.

Our analysis of disproportionality revealed that mepolizumab had significant AEs in SOCs, including Respiratory, thoracic and mediastinal disorders; General disorders and administration site conditions; Injury, poisoning and procedural complications; Infections and infestations; Surgical and medical procedures; and Social circumstances. Mepolizumab, in the context of infectious and infestations within the SOC, was frequently linked to pneumonia (n = 1,654), herpes zoster (n = 266), and lower respiratory tract infection (n = 203). The commonly reported adverse events related to respiratory, thoracic, and mediastinal disorders were dyspnea, asthma, cough, and wheezing. Notably, asthmatic crisis exhibited a strong correlation, with a significant signal strength of ROR 114.39 (103.51–126.43), PRR 113.54, IC 4.86, and EBGM 85.29. In previous clinical trials, headache and nasopharyngitis have consistently been identified as the most frequent AEs, while asthma crisis has been recognized as a significant and severe adverse event(Ortega et al., 2014; Pavord et al., 2017; Wechsler et al., 2017; Han et al., 2021). However, our study diverges in that the most prevalent adverse reactions were dyspnea, pneumonia, hospitalization, skin contact, and asthma crisis. These adverse reactions can have grave consequences. Significantly, the identical mepolizumab employed during phase III clinical studies, albeit administered at different quantities, has been associated with a heightened susceptibility to pneumonia in individuals with eosinophilic chronic obstructive pulmonary disease (Pavord et al., 2017). The main uses of mepolizumab include treating asthma, eosinophilic granulomatosis with polyangiitis, hypereosinophilic syndrome, and nasal polyps. It is worth mentioning that dyspnea, one of the recognized side effects linked to the utilization of mepolizumab in our study, could also originate from the primary illness.

Previous studies have shown that mepolizumab is primarily used for treating asthma. These studies have also identified common side effects such as headache and nasopharyngitis (Pavord et al., 2012; Khurana et al., 2019). However, our analysis has revealed a lower occurrence and weaker signals of sinus dysfunction, sinus congestion, and nasal congestion as potential side effects. The COSMEX study found that asthma worsening was the second most common negative outcome observed during mepolizumab therapy, occurring after nasopharyngitis, especially in individuals with severe eosinophilic asthma. Furthermore, asthma exacerbation emerged as the most commonly reported severe adverse incident, impacting 10% of individuals. Notably, patients who experienced treatment intervals longer than 12 weeks reported a deterioration in asthma symptoms. This highlights the potential risk of asthma exacerbation with the use or discontinuation of the monoclonal antibody. Encouragingly, the majority of clinical trials have not identified any significant adverse reactions associated with mepolizumab. Long-term monotherapy with mepolizumab appears to contribute to maintaining stable asthma control.

In our study, the most common infection type was purulent sputum, followed by helminthic infection, pharyngitis caused by fungi, allergic aspergillosis in the bronchopulmonary system, bacterial infection in the lower respiratory tract, fungal infection in the respiratory tract, and viral infection in the lower respiratory tract. Additionally, upper respiratory tract infection was also a common infection, consistent with our findings. It is important to note that asthma itself does not increase the risk of SARS-CoV-2 infection. However, it is worth mentioning that our results indicate a correlation between infections with coronaviruses not explicitly stated, such as COVID-19. It is crucial to highlight that viral infections serve as the primary risk factor for acute asthma exacerbations(Busse et al., 2010; Satia et al., 2020). An increase in ACE2 receptor expression was observed in a subset of individuals with asthma who exhibited elevated Th1 and reduced Th2 epithelial gene expression. The heightened manifestation of ACE2 receptor could potentially enhance the likelihood of negative consequences in pneumonia resulting from coronaviruses(Camiolo et al., 2020). Consistently, there was an inverse association between ACE2 gene expression and Th2 gene expression(Bradding et al., 2020). Furthermore, in a national cohort study conducted in Korea, YANG and colleagues(Yang et al., 2020) found that individuals with non-allergic asthma faced an increased likelihood of testing positive for SARS-CoV-2 and experiencing severe clinical outcomes associated with neocoronary pneumonia. Mepolizumab has the potential to modify the host immune response by inhibiting IL-5 expression, which can increase susceptibility to SARS-CoV-2 infection by suppressing Th2 responses. However, it is reassuring to highlight that the majority of clinical studies have demonstrated the safety of biologics, including mepolizumab(Cheng et al., 2004). There were notable decreases in eosinophil counts among patients receiving biologics, which were not linked to an elevated severity of neocoronaryngitis or increased mortality rates (Adir et al., 2021).Nevertheless, the observation from our study regarding the potential association between the use of mepolizumab and coronavirus infection should be taken seriously. Further investigations are warranted to assess this relationship in real-world settings.

The analysis of TTO showed that the median time for mepolizumab-related adverse events to occur was 109 days, with most cases happening within the initial month (n = 1,134, 34.75%) following mepolizumab treatment. Furthermore, we noticed a swift rise in the likelihood of AEs following the third month, eventually reaching an approximate 30% rate within a year. Moreover, the likelihood of encountering at least one worsening during the duration of the therapy rose from 24.2% (95% CI, 21.0%–27.7%) at week 16%–49.1% (95% CI, 45.2%–53.1%) at week 52, as stated in the preceding COSMOS study(Khurana et al., 2019). The findings indicated the importance of closely monitoring the AEs experienced by patients throughout the entire duration of treatment.

According to the data presented in Table 2, there was a greater occurrence of adverse drug reactions among female patients in comparison to male patients. It is essential to consider gender-biased analyses when evaluating the safety of drugs due to this observed difference in gender(Fuseini and Newcomb, 2017). To further investigate the correlation between gender and negative drug reactions, we performed gender-based subgroup analysis. According to Figure 4, it can be observed that females are more prone to encountering gastrointestinal and nervous system adverse reactions, including queasiness, bowel movements, throwing up, migraines, in addition to discomfort in the back, muscular discomfort, and muscular contractions. Infections can occur in both genders, but it is notable that pneumonia is more likely to occur in males, while influenza, bronchitis, and urinary tract infections are more common in females. Interestingly, males have a higher probability of experiencing chest pain, dyspnea, and serious adverse effects leading to hospitalization and death compared to females. Conversely, women are more frequently linked to asthmatic episodes. In order to enhance our comprehension of the correlation between gender and adverse drug reactions, we conducted additional validation of our findings through the adjustment of the *p*-values. Male patients exhibited a higher prevalence of mortality, pneumonia, heart attack, COPD, elevated blood immunoglobulin E levels, gout, decline in overall health, and cardiovascular disease in comparison to their female counterparts. On the other hand, female patients experienced a higher prevalence of headache, nausea, hair loss, pain at the injection site, back pain, fatigue, urinary tract pain, and unintentional exposure to the product. Although several clinical trials conducted in asthma, chronic rhinitis, and eosinophilic chronic obstructive pulmonary disease did not report any deaths associated with drug therapy, post-marketing data revealed that deaths accounted for 7.55 percent of serious adverse reactions, with at least 1,066 cases(Pavord et al., 2012; Chupp et al., 2017; Pavord et al., 2017; Han et al., 2021; Jackson et al., 2022). Males exhibited a higher likelihood of experiencing deaths in comparison to females. The occurrence of this could be ascribed to environmental elements like tobacco use, alcohol consumption, and other detrimental behaviors commonly seen in males, potentially resulting in coexisting conditions like pneumonia. Consequently, it reminds us that male patients undergoing treatment with mepolizumab may have a poorer prognosis. Furthermore, male patients are more susceptible to acute myocardial infarction and cardiac diseases. Although previous clinical studies did not report any drug-related serious cardiovascular AEs, it serves as a reminder to be cautious and warn about the symptoms of chest pain, especially in male patients presenting with such symptoms during the use of the drug. During a prior clinical trial examining the efficacy of mepolizumab in treating resistant eosinophilic asthma, a single instance of chest discomfort was documented in the experimental group, whereas the control group did not report any incidents of chest pain (Haldar et al., 2009). Furthermore, there was a case study detailing the occurrence of noncardiogenic chest discomfort linked to mepolizumab in a 66-year-old male individual (Korbitz et al., 2020). Earlier research has found a connection between the category of adverse events and the age at which they occur, indicating that headaches are more prevalent during the initial stages of asthma (Khatri et al., 2019). Moreover, this research contributes to the current understanding by emphasizing the correlation between the category of adverse events and gender, particularly noting that women experience headaches more frequently. Therefore, it is important to closely observe the usage of this medication in young females to detect any instances of headaches. Curiously, a female patient, aged 32, experienced hair loss after 4 months of receiving mepolizumab. The dermatology department assessed the condition as reversible alopecia caused by biologic therapy (Nixon et al., 2020). This finding aligns with our analyses, which also determined that women are more susceptible to hair loss. Moreover, as a result of the distinct physiological traits of females, infections caused by drugs mainly appear as urinary tract infections. These findings emphasize the importance of focusing on adverse reactions in clinical practice among patients of different genders. Nevertheless, it is essential to emphasize that additional clinical evidence is required to verify these findings.

To investigate and examine the adverse reaction signals linked to mepolizumab, we employed the FAERS database in our study. The method possesses robust extrapolation capability and efficiently overcomes the constraints of limited sample sizes and brief observation periods in clinical trials. Our analysis focused on AEs associated with mepolizumab, along with other pertinent and significant AEs. The objective was to offer valuable perspectives for the surveillance and improvement of clinical drug safety. Nevertheless, it is important to be aware that in spontaneous reporting systems (including FAERS), adverse event reports are voluntary and come from a variety of sources, so varying degrees of underreporting, delayed reporting, and misreporting to incomplete information may introduce bias into the measurement of the disproportionality report (Alomar et al., 2020; Khan et al., 2020; Noguchi et al., 2021). Furthermore, even when the reports are complete, it is seldom possible to enumerate the denominator or potential user population, so neither incidence nor risk can be calculated (Crisafulli et al., 2023). Finally, the signals of adverse reactions identified using the disproportionality method partially reflect the existence of a statistical correlation between a particular drug and the corresponding adverse reaction, but do not establish causality (Xia et al., 2023). Considering the above shortcomings and other potential confounders and biases, we need to interpret the results of these analyses more cautiously and further clinical study evaluations are required to confirm these associations. Although the FAERS database has its limitations in pharmacovigilance studies, our thorough analysis of the adverse event signals associated with mepolizumab and the discovery of unforeseen adverse event signals could lay the groundwork for future clinical research on this medication.

## Data Availability

Publicly available datasets were analyzed in this study. This data can be found here: All data come from the FAERS database, which is available at https://fis.fda.gov/extensions/FPD-QDE-FAERS/FPD-QDE-FAERS.html.

## Supplementary material

SUPPLEMENTARY FIGURE S1 Age-based subgroup analysis of mepolizumab-related AEs.

SUPPLEMENTARY FIGURE S2 Gender-based subgroup analysis of mepolizumab-related AEs.

SUPPLEMENTARY FIGURE S3 Weight-based subgroup analysis of mepolizumab-related AEs.

SUPPLEMENTARY FIGURE S4 Reported person-based subgroup analysis of mepolizumab-related AEs.

SUPPLEMENTARY TABLE S1 Signal strength of reports of mepolizumab at the Preferred Terms (PT) level in the FAERS database (ranked by EBGM05 value).

SUPPLEMENTARY TABLE S2 Gender-based disproportionate analysis of linezolid-related AEs. **(A)**, number of reports of target AE in female; **(B)**, number of reports of other AEs in female; **(C)**, number of reports of target AE in male; d, number of reports of other AEs in male. Therefore, the ROR here is not a strictly defined ROR in pharmacoepidemiological perspective; we just use this algorithm for signal value calculation of gender-based signal strength differences.
