# Supplementary material for: A real-world pharmacovigilance study of mepolizumab in the FDA adverse event reporting system (FAERS) database
Source: Front Pharmacol. 2023 Dec 21;14:1320458. doi: 10.3389/fphar.2023.1320458 (PMC10771301; doi:10.3389/fphar.2023.1320458)
Supplement: Supplementary file 2 [file Table1.docx]

| **SOC Name** | **Perferred terms (PTs)** | **Case Numbers** | **ROR(95%Cl)** | **PRR** | **χ2** | **IC (IC025)** | **EBGM (EBGM05)** |
| --- | --- | --- | --- | --- | --- | --- | --- |
| **Injury, poisoning and procedural complications** | Exposure via skin contact | 600 | 151.10(137.88-165.58) | 149.67 | 68217.54 | 6.85(5.18) | 115.45(106.94) |
| **Respiratory, thoracic and mediastinal disorders** | Asthmatic crisis | 474 | 114.39(103.51-126.43) | 113.54 | 43103.42 | 6.54(4.87) | 92.74(85.29) |
| **Surgical and medical procedures** | Bronchoplasty | 4 | 222.52(68.52-722.60) | 222.5 | 610.64 | 7.27(5.43) | 154.35(57.61) |
| **Investigations** | Antineutrophil cytoplasmic antibody negative | 3 | 300.40(71.79-1257.03) | 300.38 | 559.47 | 7.56(5.63) | 188.11(56.79) |
| **Surgical and medical procedures** | Patient isolation | 28 | 76.63(51.48-114.07) | 76.6 | 1811.94 | 6.06(4.38) | 66.57(47.72) |
| **Surgical and medical procedures** | Quarantine | 23 | 60.95(39.53-93.96) | 60.92 | 1208.56 | 5.77(4.09) | 54.42(37.88) |
| **Investigations** | Peak expiratory flow rate decreased | 22 | 57.99(37.29-90.17) | 57.97 | 1103.87 | 5.70(4.02) | 52.06(35.98) |
| **Infections and infestations** | Sputum purulent | 29 | 40.12(27.48-58.58) | 40.11 | 1023.8 | 5.22(3.55) | 37.21(27.11) |
| **Social circumstances** | Social problem | 116 | 33.06(27.39-39.89) | 33 | 3376.76 | 4.96(3.29) | 31.02(26.50) |
| **Investigations** | Eosinophil count abnormal | 31 | 33.11(23.02-47.62) | 33.09 | 904.96 | 4.96(3.29) | 31.10(22.94) |
| **Investigations** | Coronavirus test positive | 51 | 29.57(22.30-39.23) | 29.55 | 1328.47 | 4.81(3.14) | 27.96(22.07) |
| **Product issues** | Product complaint | 646 | 23.12(21.36-25.03) | 22.89 | 12940.72 | 4.46(2.79) | 21.94(20.53) |
| **Investigations** | Forced expiratory volume abnormal | 7 | 40.29(18.65-87.01) | 40.28 | 248.17 | 5.22(3.53) | 37.36(19.61) |
| **Investigations** | Eosinophil count decreased | 41 | 23.91(17.48-32.71) | 23.9 | 858.47 | 4.51(2.85) | 22.85(17.58) |
| **Psychiatric disorders** | Sleep disorder due to a general medical condition | 282 | 19.56(17.36-22.04) | 19.48 | 4758.68 | 4.23(2.57) | 18.78(17.00) |
| **Surgical and medical procedures** | Nasal polypectomy | 5 | 38.51(15.51-95.65) | 38.51 | 169.63 | 5.16(3.46) | 35.83(16.74) |
| **General disorders and administration site conditions** | Mucosal discolouration | 17 | 26.36(16.18-42.93) | 26.35 | 393.86 | 4.65(2.98) | 25.08(16.68) |
| **Investigations** | Peak expiratory flow rate abnormal | 4 | 41.72(15.04-115.71) | 41.72 | 146.75 | 5.27(3.56) | 38.59(16.44) |
| **Respiratory, thoracic and mediastinal disorders** | Sputum discoloured | 203 | 18.03(15.67-20.74) | 17.97 | 3141.96 | 4.12(2.45) | 17.39(15.46) |
| **Surgical and medical procedures** | Sinus operation | 45 | 19.22(14.27-25.89) | 19.21 | 747.95 | 4.21(2.54) | 18.53(14.45) |
| **Investigations** | Coronavirus test negative | 3 | 40.59(12.52-131.66) | 40.59 | 107.16 | 5.23(3.51) | 37.62(14.06) |
| **Congenital, familial and genetic disorders** | BRCA1 gene mutation | 3 | 36.63(11.34-118.31) | 36.63 | 96.89 | 5.10(3.38) | 34.20(12.82) |
| **Injury, poisoning and procedural complications** | Traumatic shock | 3 | 31.96(9.95-102.68) | 31.96 | 84.56 | 4.91(3.20) | 30.10(11.33) |
| **Investigations** | Blood immunoglobulin E increased | 36 | 13.26(9.52-18.46) | 13.25 | 397.29 | 3.69(2.03) | 12.94(9.81) |
| **Injury, poisoning and procedural complications** | Wrong technique in device usage process | 560 | 10.41(9.57-11.32) | 10.33 | 4625.01 | 3.34(1.68) | 10.14(9.45) |
| **Blood and lymphatic system disorders** | Eosinopenia | 3 | 23.47(7.37-74.70) | 23.47 | 61.64 | 4.49(2.79) | 22.46(8.52) |
| **Nervous system disorders** | Mononeuropathy multiplex | 6 | 16.78(7.44-37.86) | 16.78 | 86.16 | 4.02(2.34) | 16.27(8.24) |
| **Infections and infestations** | Helminthic infection | 5 | 17.75(7.28-43.32) | 17.75 | 76.34 | 4.10(2.42) | 17.18(8.14) |
| **Respiratory, thoracic and mediastinal disorders** | Use of accessory respiratory muscles | 6 | 16.42(7.28-37.02) | 16.41 | 84.09 | 3.99(2.31) | 15.92(8.06) |
| **Nervous system disorders** | Pachymeningitis | 5 | 17.51(7.18-42.70) | 17.5 | 75.18 | 4.08(2.40) | 16.95(8.04) |
| **Respiratory, thoracic and mediastinal disorders** | Sputum retention | 12 | 13.26(7.48-23.53) | 13.26 | 132.54 | 3.69(2.02) | 12.95(8.01) |
| **Infections and infestations** | Bronchopulmonary aspergillosis allergic | 14 | 12.63(7.43-21.47) | 12.63 | 146.22 | 3.63(1.96) | 12.34(7.92) |
| **Infections and infestations** | Fungal pharyngitis | 6 | 15.98(7.09-36.02) | 15.98 | 81.64 | 3.96(2.28) | 15.51(7.86) |
| **Investigations** | Vital capacity decreased | 8 | 13.86(6.87-27.98) | 13.86 | 92.87 | 3.76(2.08) | 13.51(7.51) |
| **Investigations** | Heart sounds | 3 | 20.03(6.32-63.50) | 20.03 | 52.14 | 4.27(2.57) | 19.29(7.35) |
| **Investigations** | Breath sounds abnormal | 47 | 9.50(7.12-12.68) | 9.5 | 350.62 | 3.22(1.56) | 9.34(7.33) |
| **General disorders and administration site conditions** | Secretion discharge | 117 | 8.43(7.02-10.12) | 8.41 | 751.95 | 3.05(1.39) | 8.29(7.12) |
| **Respiratory, thoracic and mediastinal disorders** | Pulmonary congestion | 97 | 8.51(6.97-10.41) | 8.5 | 631.61 | 3.07(1.40) | 8.38(7.08) |
| **General disorders and administration site conditions** | Idiopathic environmental intolerance | 3 | 19.26(6.08-61.01) | 19.26 | 50 | 4.22(2.52) | 18.58(7.08) |
| **Infections and infestations** | Lower respiratory tract infection bacterial | 8 | 12.29(6.09-24.78) | 12.29 | 80.95 | 3.59(1.91) | 12.02(6.68) |
| **Respiratory, thoracic and mediastinal disorders** | Bronchomalacia | 3 | 17.88(5.65-56.56) | 17.88 | 46.16 | 4.11(2.42) | 17.30(6.60) |
| **Investigations** | Blood immunoglobulin E decreased | 3 | 17.26(5.46-54.58) | 17.26 | 44.43 | 4.06(2.37) | 16.72(6.38) |
| **Investigations** | Total lung capacity decreased | 16 | 9.74(5.94-15.97) | 9.73 | 122.98 | 3.26(1.59) | 9.57(6.32) |
| **Respiratory, thoracic and mediastinal disorders** | Bronchospasm | 93 | 7.51(6.12-9.22) | 7.5 | 516.58 | 2.89(1.22) | 7.41(6.24) |
| **Respiratory, thoracic and mediastinal disorders** | Tracheomalacia | 5 | 13.39(5.51-32.54) | 13.39 | 55.81 | 3.71(2.03) | 13.06(6.21) |
| **Investigations** | Breath sounds | 3 | 16.69(5.28-52.72) | 16.69 | 42.82 | 4.02(2.32) | 16.18(6.18) |
| **Respiratory, thoracic and mediastinal disorders** | Sputum increased | 21 | 8.87(5.76-13.65) | 8.86 | 143.98 | 3.13(1.46) | 8.73(6.08) |
| **Respiratory, thoracic and mediastinal disorders** | Vocal cord dysfunction | 9 | 10.75(5.56-20.82) | 10.75 | 77.95 | 3.40(1.73) | 10.55(6.07) |
| **Investigations** | Carbon dioxide increased | 13 | 9.59(5.54-16.60) | 9.59 | 98.09 | 3.24(1.57) | 9.42(5.95) |
| **Infections and infestations** | Coronavirus infection | 78 | 7.16(5.72-8.95) | 7.15 | 406.92 | 2.82(1.15) | 7.06(5.86) |
| **Investigations** | Total lung capacity abnormal | 4 | 13.72(5.08-37.04) | 13.72 | 45.9 | 3.74(2.06) | 13.38(5.83) |
| **Investigations** | SARS-CoV-2 test negative | 11 | 9.75(5.37-17.70) | 9.75 | 84.7 | 3.26(1.59) | 9.58(5.81) |
| **Respiratory, thoracic and mediastinal disorders** | Pulmonary pain | 30 | 7.90(5.51-11.34) | 7.9 | 178.02 | 2.96(1.30) | 7.79(5.76) |
| **Congenital, familial and genetic disorders** | Factor V Leiden mutation | 5 | 12.15(5.00-29.51) | 12.15 | 49.96 | 3.57(1.89) | 11.89(5.66) |
| **Neoplasms benign, malignant and unspecified (incl cysts and polyps)** | Leiomyoma | 7 | 10.72(5.07-22.66) | 10.72 | 60.38 | 3.39(1.72) | 10.51(5.62) |
| **Musculoskeletal and connective tissue disorders** | Crystal arthropathy | 3 | 15.02(4.76-47.36) | 15.02 | 38.11 | 3.87(2.18) | 14.61(5.59) |
| **Immune system disorders** | Type III immune complex mediated reaction | 5 | 11.92(4.91-28.94) | 11.92 | 48.86 | 3.54(1.87) | 11.67(5.55) |
| **Social circumstances** | Loss of personal independence in daily activities | 472 | 6.06(5.53-6.64) | 6.02 | 1954.58 | 2.58(0.91) | 5.96(5.52) |
| **Surgical and medical procedures** | Ear operation | 4 | 12.68(4.70-34.19) | 12.67 | 41.95 | 3.63(1.95) | 12.39(5.40) |
| **Investigations** | Oxygen saturation abnormal | 24 | 7.49(5.00-11.21) | 7.49 | 132.89 | 2.89(1.22) | 7.39(5.27) |
| **Injury, poisoning and procedural complications** | Accidental exposure to product | 578 | 5.73(5.27-6.22) | 5.68 | 2209.48 | 2.49(0.83) | 5.63(5.26) |
| **Social circumstances** | Dependence on oxygen therapy | 6 | 10.43(4.65-23.41) | 10.43 | 50.11 | 3.36(1.68) | 10.24(5.20) |
| **Respiratory, thoracic and mediastinal disorders** | Increased upper airway secretion | 19 | 7.65(4.87-12.04) | 7.65 | 108.23 | 2.92(1.25) | 7.55(5.17) |
| **Infections and infestations** | Respiratory tract infection fungal | 6 | 10.29(4.58-23.09) | 10.29 | 49.29 | 3.34(1.66) | 10.10(5.14) |
| **Infections and infestations** | Respiratory tract infection | 158 | 5.88(5.02-6.88) | 5.87 | 630.69 | 2.54(0.87) | 5.81(5.09) |
| **Respiratory, thoracic and mediastinal disorders** | Increased viscosity of upper respiratory secretion | 9 | 8.78(4.54-16.98) | 8.78 | 61 | 3.11(1.44) | 8.65(4.98) |
| **Infections and infestations** | Lower respiratory tract infection viral | 6 | 9.79(4.36-21.95) | 9.78 | 46.41 | 3.27(1.59) | 9.62(4.89) |
| **Injury, poisoning and procedural complications** | Device use confusion | 4 | 11.25(4.18-30.31) | 11.25 | 36.54 | 3.46(1.78) | 11.02(4.81) |
| **Surgical and medical procedures** | Endotracheal intubation | 18 | 7.06(4.43-11.24) | 7.06 | 92.28 | 2.80(1.13) | 6.97(4.72) |
| **Surgical and medical procedures** | Enterostomy | 4 | 11.00(4.09-29.64) | 11 | 35.59 | 3.43(1.75) | 10.79(4.71) |
| **Injury, poisoning and procedural complications** | Exposure to allergen | 3 | 12.52(3.98-39.36) | 12.52 | 31.01 | 3.61(1.93) | 12.24(4.69) |
| **Infections and infestations** | Pneumonia | 1654 | 4.90(4.67-5.15) | 4.8 | 4957.5 | 2.25(0.59) | 4.76(4.57) |
| **Infections and infestations** | Chronic sinusitis | 25 | 6.40(4.32-9.50) | 6.4 | 112.52 | 2.66(1.00) | 6.33(4.55) |
| **Investigations** | Sputum abnormal | 6 | 8.91(3.98-19.98) | 8.91 | 41.42 | 3.13(1.46) | 8.77(4.47) |
| **Injury, poisoning and procedural complications** | Incision site discharge | 3 | 11.83(3.76-37.16) | 11.83 | 29.05 | 3.53(1.85) | 11.58(4.44) |
| **Respiratory, thoracic and mediastinal disorders** | Catarrh | 8 | 8.03(3.99-16.14) | 8.03 | 48.43 | 2.98(1.31) | 7.92(4.41) |
| **Infections and infestations** | Sialoadenitis | 10 | 7.43(3.98-13.87) | 7.43 | 54.81 | 2.87(1.21) | 7.33(4.35) |
| **Immune system disorders** | Multiple allergies | 46 | 5.60(4.19-7.49) | 5.6 | 171.7 | 2.47(0.80) | 5.54(4.35) |
| **Surgical and medical procedures** | Emergency care | 21 | 6.21(4.04-9.55) | 6.21 | 90.6 | 2.62(0.95) | 6.14(4.28) |
| **Respiratory, thoracic and mediastinal disorders** | Dyspnoea | 2490 | 4.60(4.42-4.79) | 4.46 | 6680.8 | 2.15(0.48) | 4.43(4.28) |
| **Surgical and medical procedures** | Polypectomy | 6 | 8.46(3.78-18.96) | 8.46 | 38.82 | 3.06(1.39) | 8.34(4.24) |
| **Infections and infestations** | Haemophilus infection | 7 | 8.04(3.81-16.96) | 8.04 | 42.45 | 2.99(1.32) | 7.93(4.24) |
| **General disorders and administration site conditions** | Nonspecific reaction | 27 | 5.89(4.03-8.60) | 5.88 | 108.21 | 2.54(0.88) | 5.83(4.24) |
| **Musculoskeletal and connective tissue disorders** | Polymyalgia rheumatica | 17 | 6.34(3.93-10.23) | 6.34 | 75.46 | 2.65(0.98) | 6.27(4.20) |
| **Respiratory, thoracic and mediastinal disorders** | Hypoventilation | 17 | 6.28(3.89-10.13) | 6.28 | 74.48 | 2.63(0.97) | 6.21(4.16) |
| **Injury, poisoning and procedural complications** | Chest injury | 16 | 6.29(3.84-10.31) | 6.29 | 70.34 | 2.64(0.97) | 6.23(4.12) |
| **Injury, poisoning and procedural complications** | Product confusion | 7 | 7.72(3.66-16.29) | 7.72 | 40.32 | 2.93(1.26) | 7.62(4.08) |
| **Investigations** | Pulmonary function test decreased | 31 | 5.51(3.86-7.84) | 5.5 | 113.01 | 2.45(0.78) | 5.45(4.06) |
| **Immune system disorders** | Atopy | 3 | 10.73(3.42-33.67) | 10.73 | 25.91 | 3.40(1.71) | 10.52(4.04) |
| **Investigations** | Full blood count abnormal | 173 | 4.62(3.98-5.37) | 4.61 | 484.74 | 2.19(0.53) | 4.58(4.04) |
| **General disorders and administration site conditions** | Symptom recurrence | 31 | 5.44(3.82-7.75) | 5.44 | 111.08 | 2.43(0.76) | 5.39(4.01) |
| **Investigations** | Emergency care examination | 5 | 8.46(3.49-20.47) | 8.46 | 32.33 | 3.06(1.38) | 8.33(3.98) |
| **General disorders and administration site conditions** | Ill-defined disorder | 219 | 4.48(3.92-5.12) | 4.47 | 584.14 | 2.15(0.48) | 4.43(3.97) |
| **Surgical and medical procedures** | Intestinal operation | 9 | 6.89(3.57-13.30) | 6.89 | 44.7 | 2.77(1.10) | 6.81(3.93) |
| **General disorders and administration site conditions** | Chest discomfort | 411 | 4.23(3.84-4.66) | 4.21 | 998.29 | 2.06(0.40) | 4.18(3.85) |
| **Infections and infestations** | Herpes zoster | 266 | 4.27(3.79-4.82) | 4.26 | 658.64 | 2.08(0.42) | 4.23(3.83) |
| **Respiratory, thoracic and mediastinal disorders** | Nasal congestion | 248 | 4.17(3.68-4.73) | 4.16 | 590.46 | 2.05(0.38) | 4.13(3.72) |
| **General disorders and administration site conditions** | Injection site paraesthesia | 9 | 6.50(3.37-12.55) | 6.5 | 41.36 | 2.69(1.02) | 6.43(3.71) |
| **Respiratory, thoracic and mediastinal disorders** | Stridor | 14 | 5.80(3.42-9.82) | 5.8 | 54.94 | 2.52(0.85) | 5.74(3.70) |
| **Respiratory, thoracic and mediastinal disorders** | Rhonchi | 8 | 6.59(3.28-13.23) | 6.59 | 37.42 | 2.70(1.03) | 6.51(3.63) |
| **Investigations** | Oxygen saturation | 3 | 9.57(3.05-29.98) | 9.57 | 22.58 | 3.23(1.55) | 9.41(3.62) |
| **Injury, poisoning and procedural complications** | Exposure to fungus | 3 | 9.51(3.03-29.79) | 9.51 | 22.41 | 3.22(1.54) | 9.35(3.59) |
| **Respiratory, thoracic and mediastinal disorders** | Upper-airway cough syndrome | 43 | 4.63(3.43-6.25) | 4.63 | 121.21 | 2.20(0.53) | 4.60(3.57) |
| **Infections and infestations** | Fungal oesophagitis | 5 | 7.56(3.13-18.29) | 7.56 | 28.05 | 2.90(1.23) | 7.46(3.57) |
| **Infections and infestations** | Lower respiratory tract infection | 203 | 4.03(3.51-4.63) | 4.02 | 457.34 | 2.00(0.33) | 4.00(3.56) |
| **Respiratory, thoracic and mediastinal disorders** | Rhinitis allergic | 21 | 5.10(3.32-7.84) | 5.1 | 68.55 | 2.34(0.67) | 5.06(3.53) |
| **Surgical and medical procedures** | Prostatectomy | 4 | 8.04(2.99-21.60) | 8.04 | 24.28 | 2.99(1.31) | 7.93(3.47) |
| **Injury, poisoning and procedural complications** | Product dose omission issue | 2170 | 3.67(3.52-3.84) | 3.58 | 4050.28 | 1.83(0.17) | 3.56(3.44) |
| **Musculoskeletal and connective tissue disorders** | Eosinophilic fasciitis | 3 | 9.05(2.89-28.34) | 9.05 | 21.09 | 3.15(1.47) | 8.90(3.43) |
| **Infections and infestations** | Sinusitis bacterial | 8 | 6.14(3.06-12.34) | 6.14 | 34.03 | 2.60(0.93) | 6.08(3.39) |
| **Infections and infestations** | Rhinitis | 37 | 4.45(3.22-6.16) | 4.45 | 98.16 | 2.14(0.48) | 4.42(3.37) |
| **Injury, poisoning and procedural complications** | Product preparation issue | 30 | 4.59(3.20-6.57) | 4.59 | 83.41 | 2.19(0.52) | 4.55(3.37) |
| **Infections and infestations** | Cellulitis orbital | 4 | 7.70(2.87-20.68) | 7.7 | 22.97 | 2.93(1.25) | 7.60(3.33) |
| **Investigations** | Spirometry abnormal | 5 | 6.99(2.89-16.90) | 6.99 | 25.32 | 2.79(1.12) | 6.91(3.30) |
| **Investigations** | Pulmonary function test abnormal | 11 | 5.46(3.01-9.89) | 5.46 | 39.62 | 2.44(0.77) | 5.41(3.29) |
| **Respiratory, thoracic and mediastinal disorders** | Choking sensation | 24 | 4.63(3.10-6.92) | 4.63 | 67.61 | 2.20(0.53) | 4.59(3.28) |
| **Surgical and medical procedures** | Palliative care | 9 | 5.72(2.96-11.03) | 5.72 | 34.64 | 2.50(0.83) | 5.66(3.27) |
| **Respiratory, thoracic and mediastinal disorders** | Chronic obstructive pulmonary disease | 186 | 3.71(3.21-4.28) | 3.7 | 364.1 | 1.88(0.21) | 3.68(3.26) |
| **Surgical and medical procedures** | Hospitalisation | 614 | 3.53(3.26-3.82) | 3.5 | 1093.89 | 1.80(0.14) | 3.49(3.26) |
| **Surgical and medical procedures** | Rest regimen | 3 | 8.58(2.74-26.87) | 8.58 | 19.76 | 3.08(1.40) | 8.45(3.25) |
| **Infections and infestations** | Pneumonia streptococcal | 6 | 6.42(2.87-14.36) | 6.42 | 27.1 | 2.67(1.00) | 6.35(3.24) |
| **General disorders and administration site conditions** | Facial discomfort | 4 | 7.44(2.77-19.98) | 7.44 | 21.99 | 2.88(1.20) | 7.35(3.22) |
| **Surgical and medical procedures** | Orthopaedic procedure | 4 | 7.36(2.74-19.76) | 7.36 | 21.67 | 2.86(1.19) | 7.27(3.18) |
| **Infections and infestations** | Pseudomonas infection | 33 | 4.23(3.00-5.96) | 4.23 | 80.64 | 2.07(0.40) | 4.20(3.15) |
| **Respiratory, thoracic and mediastinal disorders** | Sinus polyp | 4 | 7.23(2.69-19.40) | 7.23 | 21.17 | 2.84(1.16) | 7.14(3.13) |
| **Infections and infestations** | Mycobacterium avium complex infection | 12 | 5.04(2.85-8.89) | 5.04 | 38.43 | 2.32(0.65) | 5.00(3.10) |
| **Respiratory, thoracic and mediastinal disorders** | Sinus congestion | 49 | 3.91(2.95-5.18) | 3.91 | 105.18 | 1.96(0.29) | 3.88(3.07) |
| **Social circumstances** | Disease risk factor | 3 | 8.08(2.58-25.27) | 8.07 | 18.3 | 2.99(1.31) | 7.96(3.07) |
| **General disorders and administration site conditions** | Therapeutic product effect incomplete | 510 | 3.33(3.05-3.64) | 3.31 | 820.21 | 1.72(0.06) | 3.30(3.07) |
| **Investigations** | Chest X-ray abnormal | 12 | 4.93(2.79-8.71) | 4.93 | 37.26 | 2.29(0.62) | 4.89(3.04) |
| **Injury, poisoning and procedural complications** | Underdose | 306 | 3.35(2.99-3.74) | 3.33 | 497.49 | 1.73(0.06) | 3.32(3.02) |
| **Infections and infestations** | Prostate infection | 6 | 5.96(2.66-13.33) | 5.96 | 24.48 | 2.56(0.89) | 5.90(3.01) |
| **General disorders and administration site conditions** | Vaccination failure | 10 | 5.02(2.69-9.35) | 5.02 | 31.84 | 2.32(0.65) | 4.98(2.95) |
| **Investigations** | Interleukin level increased | 3 | 7.70(2.46-24.09) | 7.7 | 17.23 | 2.93(1.25) | 7.60(2.93) |
| **Respiratory, thoracic and mediastinal disorders** | Nasal obstruction | 9 | 5.08(2.63-9.80) | 5.08 | 29.19 | 2.33(0.66) | 5.04(2.91) |
| **Respiratory, thoracic and mediastinal disorders** | Sinus disorder | 76 | 3.53(2.82-4.42) | 3.53 | 136.7 | 1.81(0.15) | 3.51(2.91) |
| **Investigations** | Immunoglobulins increased | 3 | 7.62(2.44-23.84) | 7.62 | 17.01 | 2.91(1.23) | 7.52(2.90) |
| **Injury, poisoning and procedural complications** | Injection related reaction | 9 | 5.02(2.61-9.69) | 5.02 | 28.71 | 2.32(0.65) | 4.98(2.88) |
| **Infections and infestations** | Pneumonia pneumococcal | 7 | 5.41(2.57-11.39) | 5.41 | 24.88 | 2.42(0.75) | 5.36(2.87) |
| **Respiratory, thoracic and mediastinal disorders** | Painful respiration | 11 | 4.70(2.60-8.51) | 4.7 | 31.73 | 2.22(0.55) | 4.66(2.84) |
| **Surgical and medical procedures** | Oxygen therapy | 11 | 4.66(2.58-8.44) | 4.66 | 31.36 | 2.21(0.54) | 4.63(2.82) |
| **Infections and infestations** | Suspected COVID-19 | 24 | 3.95(2.64-5.90) | 3.95 | 52.43 | 1.97(0.31) | 3.93(2.80) |
| **Product issues** | Product availability issue | 74 | 3.42(2.72-4.29) | 3.41 | 125.38 | 1.76(0.10) | 3.40(2.80) |
| **Respiratory, thoracic and mediastinal disorders** | Rales | 23 | 3.98(2.64-5.99) | 3.97 | 50.81 | 1.98(0.32) | 3.95(2.80) |
| **Infections and infestations** | Sinusitis fungal | 5 | 5.84(2.42-14.09) | 5.83 | 19.8 | 2.53(0.86) | 5.78(2.76) |
| **Nervous system disorders** | Bell's palsy | 7 | 5.15(2.45-10.85) | 5.15 | 23.2 | 2.35(0.68) | 5.11(2.74) |
| **Neoplasms benign, malignant and unspecified (incl cysts and polyps)** | Lung carcinoma cell type unspecified recurrent | 4 | 6.32(2.36-16.94) | 6.32 | 17.68 | 2.64(0.97) | 6.25(2.74) |
| **Respiratory, thoracic and mediastinal disorders** | Oropharyngeal discomfort | 35 | 3.63(2.60-5.06) | 3.63 | 66.1 | 1.85(0.18) | 3.61(2.73) |
| **Surgical and medical procedures** | Cataract operation | 20 | 3.95(2.54-6.13) | 3.95 | 43.63 | 1.97(0.30) | 3.92(2.71) |
| **Infections and infestations** | Infection parasitic | 4 | 6.24(2.33-16.72) | 6.24 | 17.38 | 2.63(0.95) | 6.17(2.71) |
| **Infections and infestations** | Viral upper respiratory tract infection | 27 | 3.67(2.51-5.35) | 3.67 | 51.95 | 1.87(0.20) | 3.65(2.66) |
| **Infections and infestations** | Arthritis infective | 19 | 3.90(2.48-6.12) | 3.9 | 40.6 | 1.95(0.29) | 3.87(2.66) |
| **Investigations** | Biopsy lung | 3 | 6.95(2.23-21.73) | 6.95 | 15.08 | 2.78(1.10) | 6.87(2.65) |
| **Immune system disorders** | Allergy to arthropod sting | 3 | 6.92(2.21-21.63) | 6.92 | 14.99 | 2.77(1.10) | 6.84(2.64) |
| **Endocrine disorders** | Addison's disease | 6 | 5.15(2.31-11.52) | 5.15 | 19.87 | 2.35(0.68) | 5.11(2.61) |
| **Respiratory, thoracic and mediastinal disorders** | Nasal oedema | 7 | 4.86(2.31-10.23) | 4.86 | 21.26 | 2.27(0.60) | 4.82(2.59) |
| **Investigations** | Biopsy | 5 | 5.44(2.25-13.14) | 5.44 | 17.93 | 2.43(0.76) | 5.39(2.58) |
| **Investigations** | Biopsy bone marrow | 3 | 6.71(2.15-20.95) | 6.7 | 14.37 | 2.73(1.05) | 6.63(2.56) |
| **Nervous system disorders** | Anosmia | 35 | 3.38(2.42-4.71) | 3.37 | 58.11 | 1.75(0.08) | 3.36(2.54) |
| **Cardiac disorders** | Eosinophilic myocarditis | 4 | 5.86(2.19-15.69) | 5.86 | 15.92 | 2.54(0.86) | 5.80(2.54) |
| **Injury, poisoning and procedural complications** | Abdominal injury | 3 | 6.59(2.11-20.58) | 6.59 | 14.03 | 2.70(1.03) | 6.51(2.51) |
| **Investigations** | Sputum culture positive | 3 | 6.53(2.09-20.40) | 6.53 | 13.87 | 2.69(1.01) | 6.46(2.49) |
| **Respiratory, thoracic and mediastinal disorders** | Upper respiratory tract congestion | 11 | 4.12(2.27-7.45) | 4.12 | 25.74 | 2.03(0.36) | 4.09(2.49) |
| **Respiratory, thoracic and mediastinal disorders** | Nasal septum deviation | 8 | 4.39(2.19-8.81) | 4.39 | 20.77 | 2.13(0.46) | 4.36(2.44) |
| **Infections and infestations** | Gastric infection | 16 | 3.69(2.26-6.04) | 3.69 | 31.19 | 1.88(0.21) | 3.67(2.43) |
| **Investigations** | Forced expiratory volume decreased | 15 | 3.72(2.24-6.18) | 3.72 | 29.56 | 1.89(0.22) | 3.70(2.42) |
| **Investigations** | Respiratory rate increased | 26 | 3.34(2.27-4.92) | 3.34 | 42.37 | 1.73(0.07) | 3.33(2.41) |
| **Immune system disorders** | Perfume sensitivity | 4 | 5.49(2.05-14.70) | 5.49 | 14.52 | 2.44(0.77) | 5.44(2.38) |
| **Infections and infestations** | Infected cyst | 6 | 4.69(2.10-10.49) | 4.69 | 17.28 | 2.22(0.55) | 4.66(2.38) |
| **Gastrointestinal disorders** | Salivary gland enlargement | 3 | 6.21(1.99-19.38) | 6.21 | 12.94 | 2.62(0.94) | 6.14(2.37) |
| **General disorders and administration site conditions** | Administration site pain | 6 | 4.67(2.09-10.44) | 4.67 | 17.15 | 2.21(0.54) | 4.64(2.37) |
| **Infections and infestations** | Uterine infection | 3 | 6.18(1.98-19.30) | 6.18 | 12.87 | 2.61(0.94) | 6.12(2.36) |
| **Infections and infestations** | Mastitis | 9 | 4.10(2.13-7.90) | 4.1 | 20.92 | 2.03(0.36) | 4.07(2.35) |
| **Investigations** | Computerised tomogram abnormal | 7 | 4.39(2.08-9.23) | 4.39 | 18.14 | 2.12(0.45) | 4.36(2.34) |
| **Infections and infestations** | Gingival abscess | 4 | 5.37(2.00-14.38) | 5.37 | 14.07 | 2.41(0.74) | 5.32(2.33) |
| **Gastrointestinal disorders** | Odynophagia | 18 | 3.45(2.17-5.48) | 3.45 | 31.04 | 1.78(0.11) | 3.43(2.33) |
| **Skin and subcutaneous tissue disorders** | Hand dermatitis | 3 | 6.01(1.92-18.76) | 6.01 | 12.37 | 2.57(0.90) | 5.95(2.29) |
| **General disorders and administration site conditions** | Polyp | 23 | 3.24(2.15-4.89) | 3.24 | 35.43 | 1.69(0.02) | 3.23(2.29) |
| **Infections and infestations** | Mycobacterial infection | 8 | 4.05(2.02-8.13) | 4.05 | 18.26 | 2.01(0.34) | 4.03(2.25) |
| **Respiratory, thoracic and mediastinal disorders** | Sinonasal obstruction | 3 | 5.89(1.89-18.39) | 5.89 | 12.04 | 2.54(0.87) | 5.83(2.25) |
| **Surgical and medical procedures** | Coronary arterial stent insertion | 10 | 3.80(2.04-7.08) | 3.8 | 20.49 | 1.92(0.25) | 3.78(2.25) |
| **Skin and subcutaneous tissue disorders** | Nail bed disorder | 3 | 5.87(1.88-18.31) | 5.87 | 11.97 | 2.54(0.86) | 5.81(2.24) |
| **General disorders and administration site conditions** | Local reaction | 4 | 5.12(1.91-13.72) | 5.12 | 13.13 | 2.34(0.67) | 5.08(2.23) |
| **Surgical and medical procedures** | Suture insertion | 3 | 5.82(1.87-18.17) | 5.82 | 11.84 | 2.53(0.85) | 5.77(2.22) |
| **Vascular disorders** | Arteriovenous fistula | 3 | 5.75(1.84-17.96) | 5.75 | 11.65 | 2.51(0.84) | 5.70(2.20) |
| **Injury, poisoning and procedural complications** | Traumatic lung injury | 8 | 3.87(1.93-7.77) | 3.87 | 16.92 | 1.95(0.28) | 3.85(2.15) |
| **Investigations** | Antineutrophil cytoplasmic antibody positive | 3 | 5.58(1.79-17.42) | 5.58 | 11.16 | 2.47(0.79) | 5.53(2.14) |
| **General disorders and administration site conditions** | Crepitations | 11 | 3.49(1.93-6.32) | 3.49 | 19.41 | 1.80(0.13) | 3.47(2.11) |
| **Cardiac disorders** | Cardiovascular symptom | 3 | 5.52(1.77-17.23) | 5.52 | 10.99 | 2.45(0.78) | 5.47(2.11) |
| **Investigations** | Body temperature abnormal | 9 | 3.66(1.90-7.05) | 3.66 | 17.28 | 1.86(0.20) | 3.64(2.10) |
| **Gastrointestinal disorders** | Tongue pruritus | 4 | 4.83(1.80-12.92) | 4.83 | 12.01 | 2.26(0.59) | 4.79(2.10) |
| **Infections and infestations** | Pneumonia fungal | 15 | 3.20(1.93-5.32) | 3.2 | 22.6 | 1.67(0.01) | 3.19(2.09) |
| **Respiratory, thoracic and mediastinal disorders** | Nasal inflammation | 5 | 4.35(1.81-10.50) | 4.35 | 12.8 | 2.11(0.44) | 4.32(2.07) |
| **Infections and infestations** | Ophthalmic herpes zoster | 8 | 3.71(1.85-7.43) | 3.7 | 15.68 | 1.88(0.21) | 3.69(2.06) |
| **Respiratory, thoracic and mediastinal disorders** | Restrictive pulmonary disease | 5 | 4.32(1.79-10.41) | 4.32 | 12.63 | 2.10(0.43) | 4.29(2.05) |
| **Surgical and medical procedures** | Lung operation | 4 | 4.67(1.74-12.50) | 4.67 | 11.42 | 2.21(0.54) | 4.63(2.03) |
| **Respiratory, thoracic and mediastinal disorders** | Sinus pain | 11 | 3.34(1.85-6.05) | 3.34 | 17.95 | 1.73(0.07) | 3.33(2.03) |
| **Surgical and medical procedures** | Cardiac ablation | 7 | 3.78(1.80-7.95) | 3.78 | 14.21 | 1.91(0.24) | 3.76(2.02) |
