# Supplementary figures and images for: A real-world pharmacovigilance study of mepolizumab in the FDA adverse event reporting system (FAERS) database
Source: Front Pharmacol. 2023 Dec 21;14:1320458. doi: 10.3389/fphar.2023.1320458 (PMC10771301; doi:10.3389/fphar.2023.1320458)

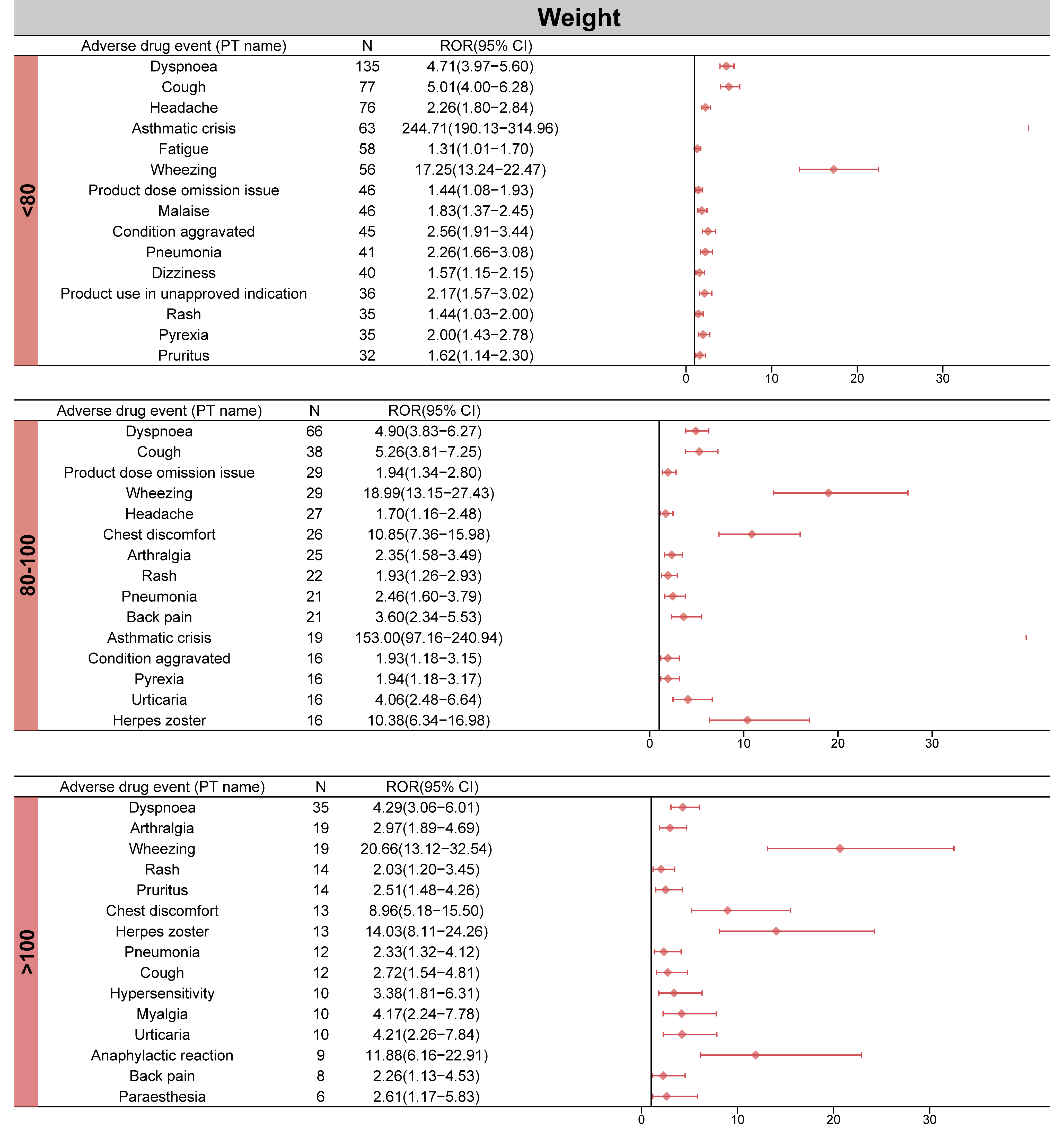

Supplement: Supplementary file 3 [file Image3.TIF]

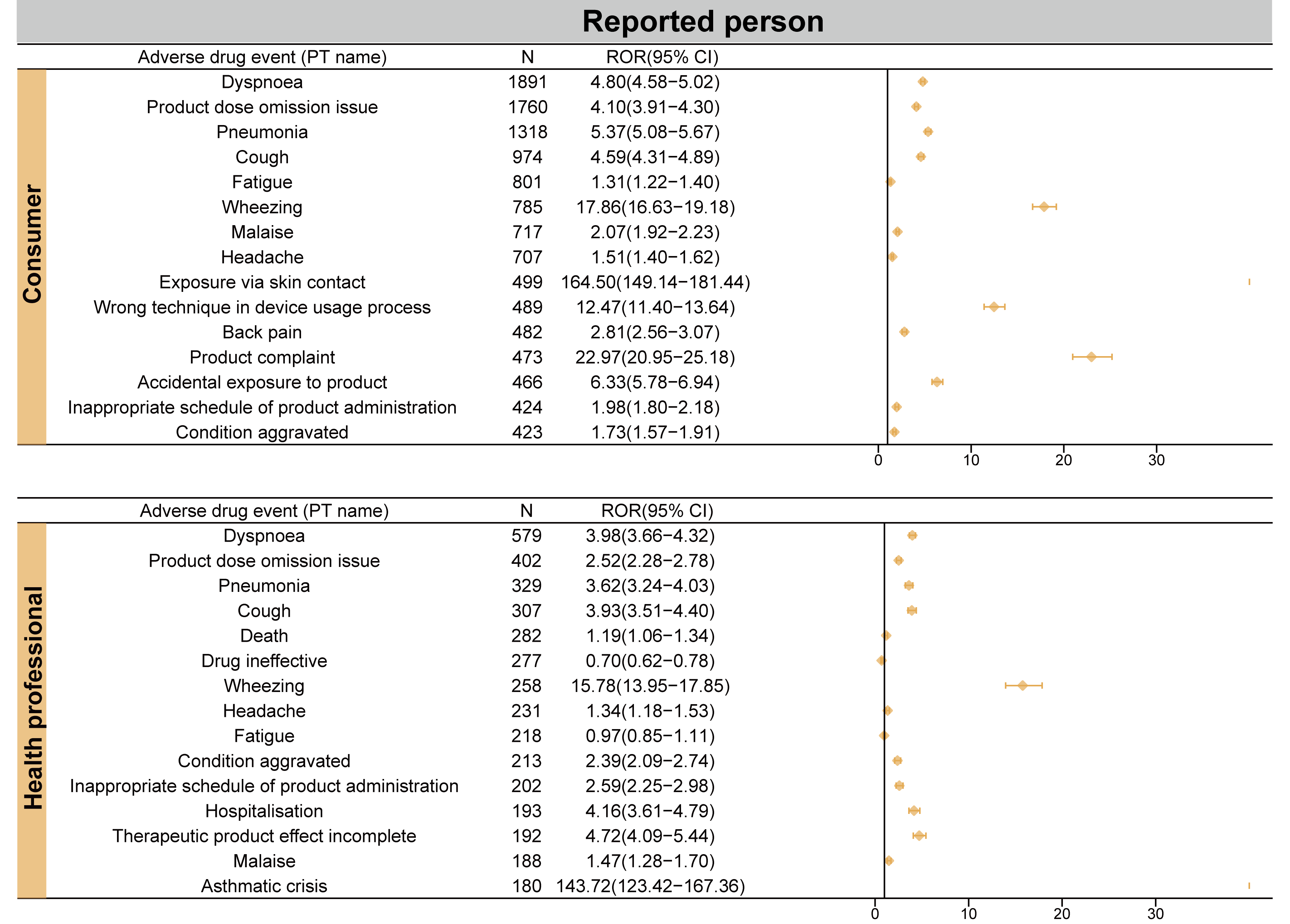

Supplement: Supplementary file 4 [file Image4.TIF]

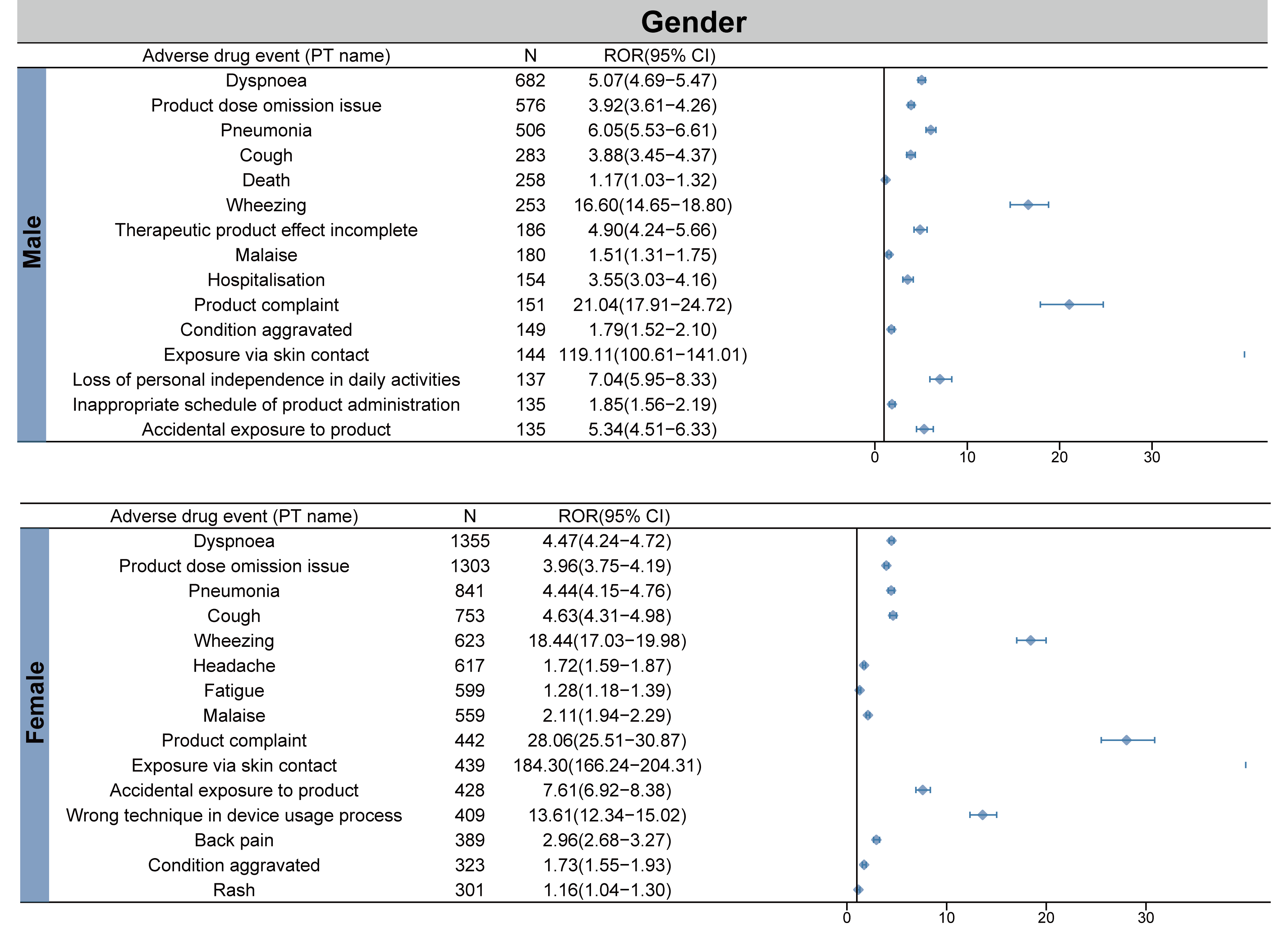

Supplement: Supplementary file 5 [file Image2.TIF]

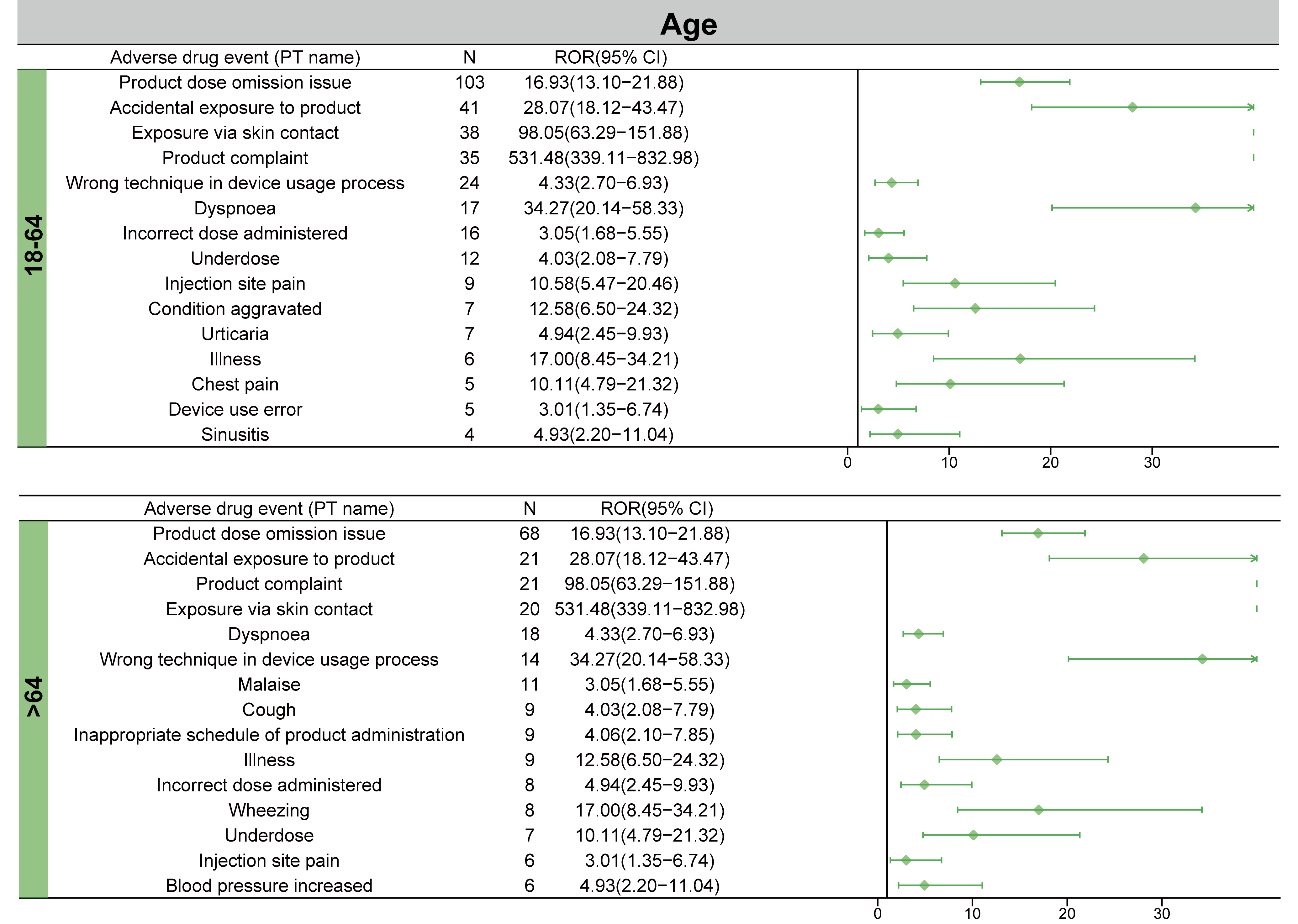

Supplement: Supplementary file 6 [file Image1.TIF]
